# Evaluating multiannual sedimentary nutrient retention in agricultural two-stage channels

**DOI:** 10.1038/s41598-024-84956-2

**Published:** 2025-01-03

**Authors:** Kaisa Västilä, Tom Jilbert

**Affiliations:** 1 Department of Built Environment, Aalto University School of Engineering, Espoo, Finland; 2 Marine and freshwater solutions unit, Finnish Environment Institute, Helsinki, Finland; 3 Environmental Geochemistry group, Department of Geosciences and Geography, University of Helsinki, Helsinki, Finland

**Keywords:** Two-stage channels, Sedimentary nutrients, Deposition, Suspended sediment, Phosphorus, Floodplains, Biogeochemistry, Environmental sciences, Hydrology, Engineering

## Abstract

The two-stage channel (TSC) design with a vegetated man-made floodplain has been recommended as an alternative to conventional re-dredging for managing suspended sediment (SS) and nutrient loads in agricultural streams. However, there are currently uncertainties surrounding the efficiency of TSCs, since mass balances covering the whole annual hydrograph and including different periods of the channel life cycle are lacking. This paper aims to improve understanding of the medium-term morphological development and sedimentary nutrient retention when a dredged, trapezoidal-shaped channel is converted into a TSC, using a mass balance estimate of nutrient and carbon retention from immediately after excavation until the establishment of approximate biogeochemical equilibrium retention. We developed a framework allowing estimation of the sedimentary net retention of phosphorus (P), nitrogen (N) and carbon (C) considering the differences in the initial and mature biogeochemical conditions in topsoil between different channel parts. Further, we conducted repeated elevation surveys and analyses of vertical sedimentary elemental chemistry over a 9-year period to apply the framework at a pilot site in Southern Finland. The pilot TSC floodplain significantly retained SS and nutrients while the low-flow channel did not suffer from siltation, supporting the hypothesized enhanced self-cleansing capacity of TSCs compared to trapezoidal cross-sections. Because of the flushing of the earlier bed deposits, there was net release of SS, P, and N over the first 9 years in the entire TSC system. Depending on the element and channel part considered, physical deposition constituted 13‒79% of the net retention on the newly exposed, excavated surfaces, while the remainder could be attributed to biogeochemical retention, enriching the topsoil in nutrients and carbon. The developed framework is highly suitable to assess the medium-term sedimentary nutrient retention in TSC systems. As a novelty, the framework improves the reliability of the retention efficiency evaluation compared to the typically used snapshot water quality sampling and allows prioritizing the required sediment coring at further TSC sites. The results allow heterogeneities in the process rates to be quantified and potential inefficiencies in nutrient retention due to channel design and morphology to be identified.

**Supplementary Information:**

The online version contains supplementary material available at 10.1038/s41598-024-84956-2.

## Introduction

Nature-based Solutions (NbS) are increasingly recommended as a means to combine the human uses of rivers and ecological requirements for improved biodiversity and water quality^1,2^. Currently, river–floodplain systems are among the most threatened hydro-environments both in Europe and globally because of extensive hydraulic engineering, agricultural expansion and intensification, climate change, pollution, invasive species, water withdrawals, fragmentation and flow regulation^3–5^. The conventional channelization of streams and rivers into trapezoidal cross-sections, including frequent re-dredging of deposited sediment and removal of vegetation, has deteriorated riverine habitats, biodiversity and ecosystem services^6^. The associated reduction in the channels’ capacity to retain and process particulate and soluble substances has increased the downstream transport of suspended sediment (SS) and nutrients, mainly phosphorus (P) and nitrogen (N)^7^, contributing to large-scale eutrophication of surface waters^8–10^.

One promising nature-based solution for flood and agricultural water management in lowland areas are two-stage (compound) channels consisting of a narrow main (low-flow) channel and a confined floodplain excavated on one or both sides of the low-flow channel^11–13^. While vegetated floodplains of two-stage channels (TSCs) trap suspended sediment and particulate phosphorus^8,9^and remove nitrogen via denitrification^14,15^, there are major uncertainties in the total water quality impacts of TSCs^16,17^. Based on the limited comparative evidence available, TSCs provide certain benefits for aquatic and riparian biodiversity^16,18^and channel stability^19,20^while maintaining the flow conveyance and drainage depth^19,21^better than the conventional re-dredging of a channel to a trapezoidal-shaped cross-section with a wide bed. TSCs are hypothesized to have up to 3 times longer life cycles than conventionally dredged channels^22^because the narrower low-flow channel is expected to maintain higher flow velocities during low-to-medium flows, and thus to decrease siltation and excessive vegetation growth on the channel bed, which are typical problems in trapezoidal channels with wide beds^23^. Presently, field studies of the medium to long-term morphological development of agricultural TSCs are limited^17,20^while the sediment transport dynamics of such vegetated rehabilitated channels are difficult to predict numerically^24^.

The uncertainties in the efficiency of TSCs in decreasing the SS, P, and N loads stem firstly from the fact that only few works provide an experimentally obtained annual or multi-annual mass balance for these substances in TSCs^25–27^. Secondly, even fewer studies have monitored the channel from the beginning of its life cycle, i.e. straight after the excavation of the floodplain, although the large areas of bare soil may initially increase the loads^28,29^. Thirdly, most studies have investigated the changes in instantaneous concentrations using grab water sampling methodology but not considered the effects of the TSC design on the transported loads. Results on the influence of agricultural TSCs on particularly the phosphorus and suspended sediment concentrations are inconsistent. Compared to trapezoidal reference reaches, TSCs have decreased^25,30^or had a varied influence^31^on turbidity, and increased^30^or decreased^26^suspended sediment concentration (SSC). Two-sided TSCs have decreased soluble reactive phosphorus (SRP) concentration and load^27^, but in another study they only decreased the SRP concentration^25^. On the other hand, a modeling study^32^demonstrated a notable decrease in TP with two-stage channel application. Besides site-specific factors, this variability may be explained by the grab water sampling being typically biased towards baseflows^8,27,33^whereas up to 90% of the loads can be transported at floodplain flows during high discharge events^34^. Grab water sampling is prone to large uncertainties caused by the quick passage of the peak loads^35^and the difficulty in obtaining cross-sectionally representative concentrations and loads^36^. Thus, there is a pressing need for assessment methods reliably taking into account also the high flow events^37^.

To optimize the water quality benefits of TSCs, it is critical to improve understanding of the channel processes and factors that control the transport, deposition and transformations of nutrients in such managed hydro-environments. TSCs may notably differ from natural or restored floodplains^38,39^where inundation is less frequent and flows are less flashy with slower changes in e.g. redox conditions in the upper soil horizons. The retention, release and processing of nutrients within channel sediments are of major importance for the phosphorus and nitrogen budgets^40,41^, particularly in smaller channels (mean discharges of up to few m^3^/s) where the sediment-water interface area in relation to the total water volume is high. Since most of the phosphorus in fine-grained agricultural catchments is typically particle-bound^42,43^, the deposition and erosion of suspended sediment are expected to be particularly important for the P mass balance^26^. There remains a gap in knowledge on the role of the floodplains, banks and the low-flow channel of TSCs in contributing to the total mass balance of SS and the associated sedimentary retention of P, as well as other bio-active elements such as nitrogen (N) and carbon (C). Such spatially distributed information can aid in improving the design, construction and maintenance of two-stage channels by revealing how the complex flow and mixing processes control and limit the transfer of SS and nutrients across the channel cross-section^36^.

The aim of this paper is to improve understanding of the medium-term sediment transport and sedimentary nutrient retention when a conventionally dredged, trapezoidal-shaped channel is converted into a TSC. The objectives are (1) to determine the spatial distribution of net deposition and erosion of fine cohesive sediment in a pilot two-stage channel during 9 years after floodplain excavation, (2) to develop a framework allowing estimation of the sedimentary net retention of phosphorus (P), nitrogen (N) and carbon (C) in TSCs considering the differences in initial and mature biogeochemical conditions between different parts of the channel cross-section, and (3) to validate and parameterize the nutrient retention framework for estimating the depositional and biogeochemical fluxes, total mass balance and the efficiency in retaining the total loads at the case study site in Southern Finland in the first 9 years after the TSC construction.

## Site and methodology

### Study catchment and two-stage channel

The study site is a small (10.3 km^2^) headwater catchment in Sipoo, Southern Finland (Supplementary Figures S1 and S2 online). The catchment drains into a network of low-lying river systems and eventually into the Baltic Sea. Land use in the studied catchment consists of agriculture (11.7%), forests, heaths and rock (80.5%), constructed areas (4.9%), and water areas, wetlands and fens (3.0%). The Ritobäcken two-stage channel (outlet located at 60°20’05.1”N, 25°13’14.2”E) was constructed in February 2010 by excavating an 820 m long floodplain on one side of the existing main channel at the downstream part of this catchment^12^. The TSC replaced a trapezoidal drainage ditch, itself a modification of a natural brook. Prior to the TSC excavation, the conveyance capacity of the ditch had deteriorated, attributable to siltation, vegetation growth and compaction of field soil^44^, resulting in frequent flooding of the fields. The constructed TSC comprises 11.9% of the length of the main channel in the headwater catchment upstream from its outlet.

**Fig. 1:**
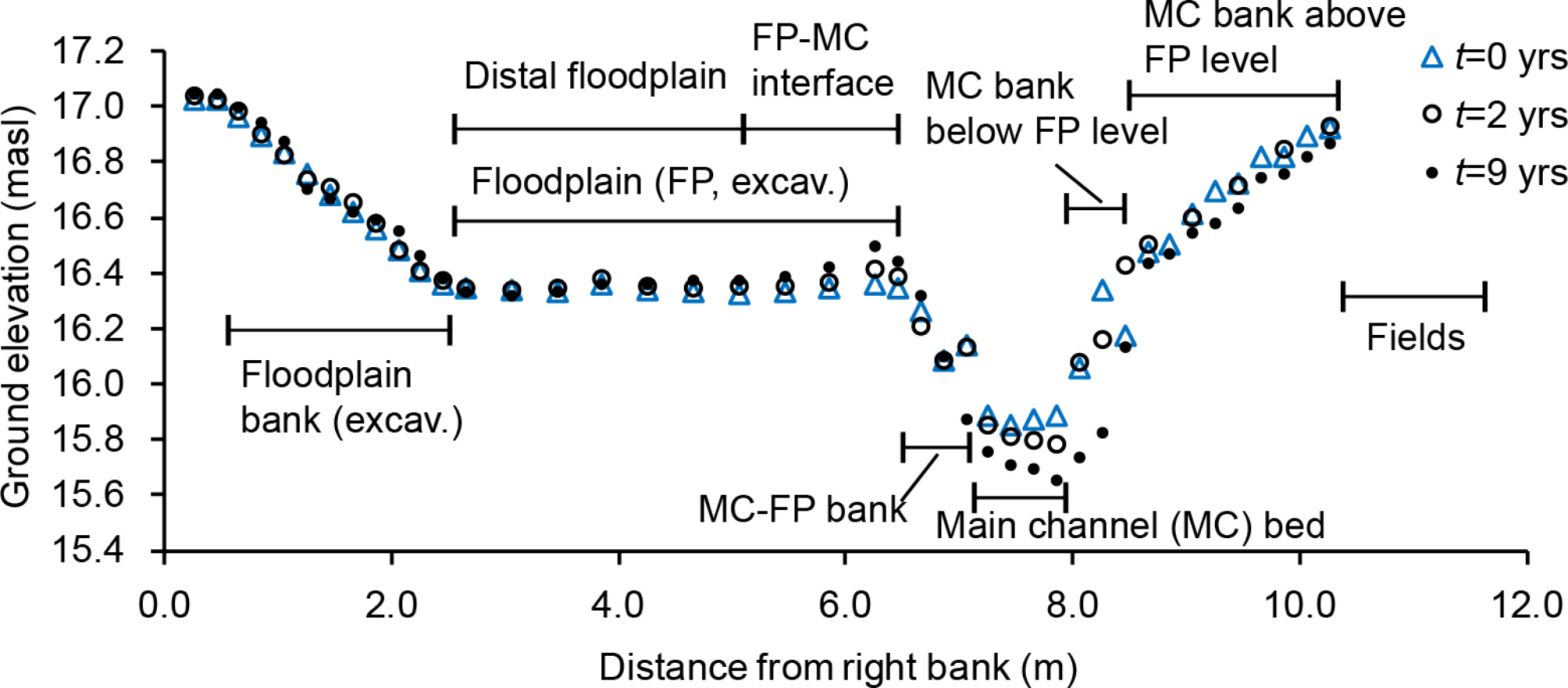
Representative two-stage channel cross-section with the cross-sectional geometry measured at *t* = 0, 2 and 9 years after the floodplain excavation, and the delineation and abbreviations of the different channel parts according to the *t* = 0 years survey.

The floodplain of the TSC was constructed at the level of the estimated mean discharge, with the floodplain bank sloping at 1:2 (Fig. 1). The rest of the cross-section, including the low-flow channel and the well-established grassy vegetation in the unexcavated channel parts, was left undisturbed (Supplementary Fig. S1 online). The two-stage cross-section extended ~ 3 m onto the former vegetated buffer strip. The mean discharge is ~ 0.12 m^3^/s, the longitudinal bed slope is 0.001–0.002, and the cross-sectional mean velocities range at 0.1–0.3 m/s^34^. As there are no sub-surface drainage pipes discharging onto the floodplain and the surface runoff from the surrounding fields is limited, we assume that particulate materials are distributed to the floodplain and floodplain banks primarily through transport from the main channel. Dissolved components may be retained either by uptake from main channel flow, or from subsurface flow of groundwaters within the floodplain and floodplain banks. Suspended sediment entering the TSC comprises on average 40% clay (particles finer than 2 μm) while the remainder is silt (2–60 μm)^34^.

The discharges (*Q*) and loads of suspended sediment (*Q*_*SS*_), total phosphorus (*Q*_*TP*_), total nitrogen (*Q*_*TN*_) and total organic carbon (*Q*_*TOC*_) entering into the two-stage channel were estimated by the WSFS-VEMALA model. When validated against continuous monitoring data^34^ and water samples, the model was found to provide reliable estimates at the monthly scale for all the other parameters except *Q*_*SS *_ (Supplementary text S1, Table S1, and Figure S3 online). Thus, additional scaling was conducted for *Q*_*SS*_, resulting in improved estimates of monthly values. The variation in the annual suspended sediment and nutrient inputs to the channel is associated mainly with variations in the mean discharge (Supplementary Figure S4 online).

We computed annual averages of various properties of the floodplain flow (Supplementary text S2 online), of which inundation duration and annual floodplain discharge were considered the most important for controlling the retention of suspended sediment and sedimentary nutrients on the floodplain. The data allows comparing the periods *t* = 0‒2 yrs and *t* = 2‒9 yrs, over which the net deposition and erosion were monitored (next section). The second period had a ~ 30% lower annual total discharge conveyed on the floodplain and ~ 10% lower annual inundation duration (Supplementary Table S2 online).

### Monitoring of cross-sectional geometry and sediment mass balance

To estimate net erosion and sediment deposition, repeated high-resolution elevation surveys were conducted at *t* = 0 and 2 years after the TSC construction (in 2010 and 2012) in five variably vegetated 20 m long sub-reaches of a 190 m-long study reach at the downstream end of the Ritobäcken TSC^34^. Six of the 10 cross-sections were appropriate for the present investigations and were re-surveyed at *t* = 9 yrs (2019, see Supplementary Figure S2 online). We excluded cross-sections in a sub-reach planted with willows, because the willow roots prevented sediment coring, and those in a sub-reach maintained bare at *t* = 0‒2 yrs, because these were influenced by large, localized floodplain deposits generated by a small side-channel discharging onto the floodplain. Natural grassy vegetation took over on most of the excavated surfaces within ~ 2‒3 years.

All cross-sectional surveys were conducted in summer when the floodplain soil was at its driest to minimize the effect of soil swelling. A plumb line on a custom-built framework fixed to steel poles at both ends of the cross-sections was used at *t* = 0‒2 yrs^34^, and a millimeter-level-accuracy total station for the surveys conducted as part of this research at *t* = 9 yrs. During all campaigns, the ground elevation was measured at a spacing of 20–40 cm by carefully pushing aside the living vegetation. The measurements were locally geo-referenced to permanent markers drilled into nearby bedrock outcrops. The accuracy of the elevation measurements varied according to the measurement instrument and the channel part (Supplementary text S3 online). The associated uncertainty was typically substantially lower than the mean 9-year net change, except for the floodplain bank where it was on average half of the mean net change.

A mass balance computation was performed to estimate net erosion and deposition of sediment in different channel parts (Fig. 1) based on laterally interpolated changes in soil surface elevation *ΔZ*. The mean dry sediment bulk density *ρ* = 475 kg/m^3^ determined from the post-construction floodplain deposits (next section) was assumed representative of floodplain and bank deposits originating after the modifications of the channel. For the eroding MC bed characterized by intermittent coarser layers, we used *ρ* = 663 kg/m^3^ derived from the MC sediment cores collected prior to floodplain excavation. The total retention of suspended sediment in the entire two-stage channel reach was obtained by multiplying the average cross-sectional retention by the length of the TSC. The 6 cross-sections were well representative of the approximately constant cross-sectional geometry in the entire two-stage reach.

### Sediment sampling for nutrient analyses

Sediment and soil cores were collected in or close to the monitored cross-sections from the permanently inundated main channel bed (*N* = 3), from the floodplain at different distances from the FP‒MC interface to cover locations with differing *ΔZ* (*N* = 9), and from the main channel bank above floodplain level (*N* = 3, Supplementary Fig. S2 online). Most of the cores were sampled in summer‒early autumn at *t*= 9 yrs with an AMS split soil core sampler (sample diameter 48 mm) while one test core collected from each of the three channel parts in November 2018 was included into the analyses. For the non-saturated floodplain and channel bank cores, the sampler was lined with plastic 13–51 mm long liners taped together to aid in accurately slicing the cores with a small fretsaw^45^. For the saturated main channel cores, the sampler was lined with an intact long liner, and the cores were sliced to 10–20 mm sections using Pylonex HTH sediment corer equipment with house-made fittings. Of the three test cores in 2018, the floodplain and bank were sampled with an Eijkelkamp piston sampler and the main channel bed with a plastic sediment tube (diameter 76 mm).

All cores were sliced on the sampling day in the field or the following day in a laboratory. The sampled material was kept in a coolbox during the field day and placed in a refrigerator at the end of the day. The three test cores were wet-sieved to separate the fractions < 63 μm and > 63 μm. The samples were dried at 45 °C for a minimum of 48 h, which was sufficient to reach a stable weight (no further loss of water). The refrigerated samples were put to the oven 1‒2 days after their collection while the samples from the test cores were kept frozen prior to drying. Additionally, we used dried samples of two comparable main channel sediment cores collected in the summers before (2009)^44^ and *t* = 2 years after (2012) channel construction. The dry bulk density *ρ* of each slice was determined by dividing the dried sediment mass by the known bulk sampling volume.

Most of the dried samples were ground with an electrical IKA A 11 basic analytical mill. Manual grinding with a pestle and mortar was used for several bulk samples as well as for the < 63 μm fraction of the sieved samples; concentrations of the manually ground samples were corrected to be comparable to the electronically ground samples (Supplementary text S4 online). Total C and N were determined by thermal combustion elemental analysis (TCEA). Total P contents were determined by ICP-OES analysis of digested samples (extracted on a high throughput hotplate with concentrated HNO_3_at 160 °C for 30 min., based on method 3050B from U.S Environmental Protection Agency^46^), and subsequent dilution of the extracted samples to 1 M HNO_3_. The method is considered to extract oxides, organic matter and reactive minerals, although a small fraction of P may remain in the residue^47^. Sulfur (S) was also determined by ICP-OES to aid with stratigraphic interpretation of the core profiles. Reproducibility of the TCEA and ICP-OES elemental analyses was 1.3‒2.6% (Supplementary text S4 online). For feasibility reasons, C and N contents were determined for three cores per each channel part, while P and S contents were determined for all cores.

### Framework for estimating the net sedimentary retention of nutrients after the two-stage channel construction

We propose a robust framework for estimating the medium-term net sedimentary retention/release of phosphorus, nitrogen and carbon in a two-stage channel between channel construction (*t =* 0) and some future moment (*t =*T) several full years later to exclude seasonal differences^8,48^. It is targeted for fine-grained sediments and gentle slopes typical of most agricultural areas for which TSCs are recommended^1^. The framework is not intended to differentiate between the flow pathways of the substances between the surrounding catchment and the channel.

**Fig. 2:**
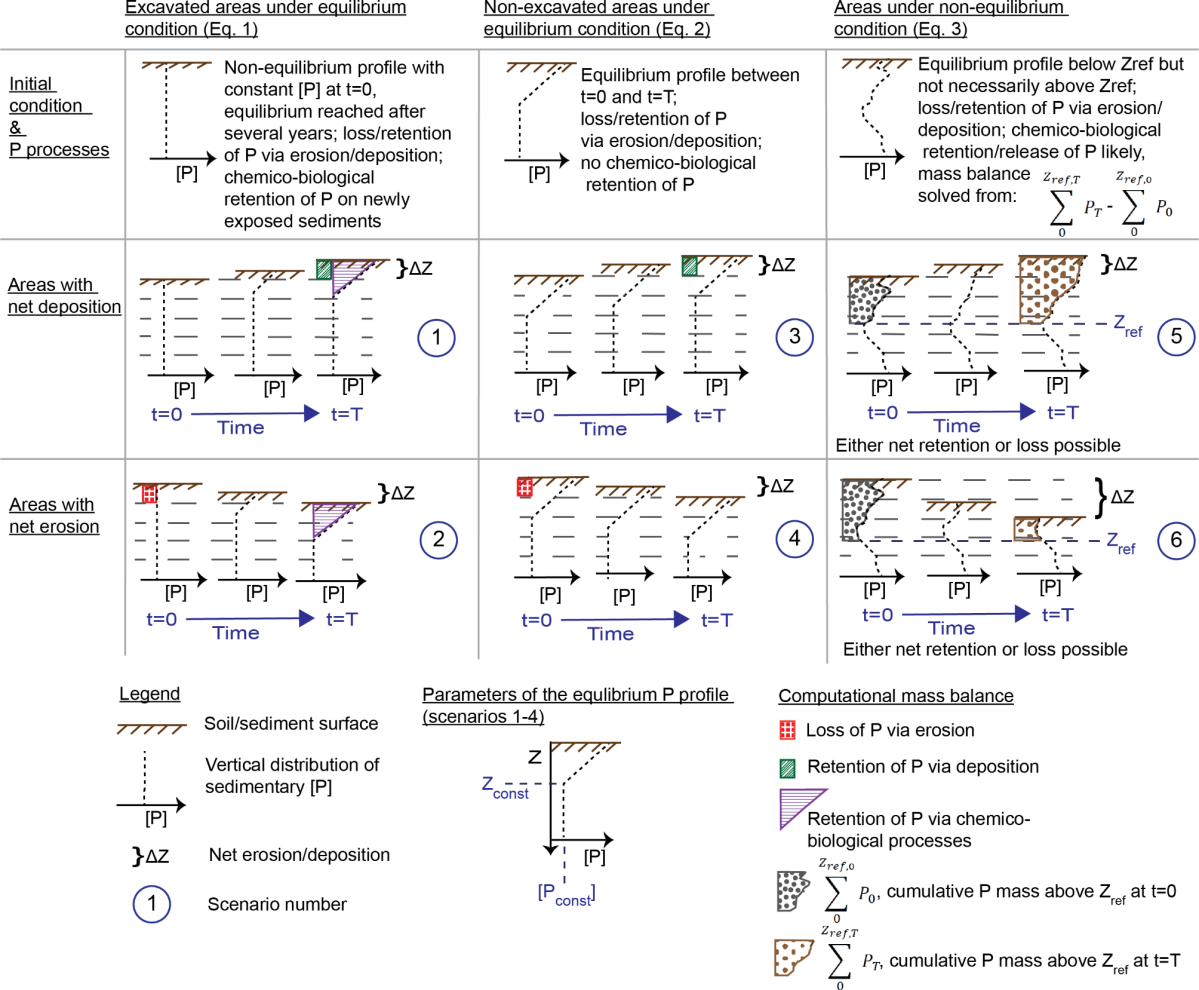
Framework for estimating the net sedimentary retention of phosphorus in low-gradient channels between the initial time point (*t* = 0) and the last time point (*t* = T) based on the temporal development of the vertical [P] profile under six different scenarios.

Each channel part is allocated to one of 6 scenarios describing the medium-term evolution of nutrient profiles in the upper decimeters of the soil after the TSC construction (Fig. 2). Three scenarios (1, 3, 5) are envisioned for areas experiencing net sedimentation, and a further three (2, 4, 6) for areas with net erosion since construction of the TSC. Scenarios 1 and 2 describe a situation in which an excavated surface has by *t =* T reached approximate equilibrium with respect to biogeochemical nutrient retention, indicated by a stable surface enrichment of P, N and C in the topsoil. Scenarios 3 and 4 describe a situation in which an unexcavated surface displays a similar equilibrium. Scenarios 5 and 6 then describe a situation in which no such equilibrium exists, and the subsurface concentrations of P, N and C are variable due to non-steady state depositional conditions. Full equations for calculation of total P, N and C retention under each scenario are given in Supplementary text S5 online. Below, we justify the overall framework.

**Scenarios 1 and 2** for excavated areas close to biogeochemical equilibrium are expected to apply for areas located well above the annual low water table, allowing oxygen penetration into the whole topsoil, and having approximately vertically uniform physical properties of the parent sediment. Biogeochemical equilibrium is expected to have been reached within a decade after the excavation of TSC floodplains, which typically have developed a rich ecosystem with abundant vegetation within such a time frame^18,49^.

We assume that initially after excavation, soil nutrient contents are low and constant, representing mainly forms of P, N and C with low reactivity in the exposed deeper horizons (Fig. 2). Excavation may remove up to several meters of soil, so it is expected that surface enrichments of P, N and C that are usually observed in the uppermost ~ 20–70 cm in vegetated soils^50–52^are completely removed. However, given time these enrichments can return. This is primarily due to deposition of organic material at the soil surface and subsequent mineralization in the soil column^8^, associated mainly with the life cycle of grassy vegetation in agricultural TSC floodplains where woody vegetation is typically not allowed^19,53^. The enrichment may occur in a context of net soil deposition (scenario 1) or minor net erosion (scenario 2). Additional processes contributing to surface enrichments include sorption of reactive P^54^and ammonium-nitrogen^55^on the fine-grained surface sediments newly exposed to nutrient-rich channel water. Net P sorption is optimal at the well-oxygenated sediment‒water interface^8^due to the presence of oxide mineral surfaces, while ammonium sorption may be promoted by high nitrate concentrations in agricultural runoff and the riparian sediments being hotspots for dissimilatory nitrate reduction to ammonia (DNRA)^48,56^. The processes influencing the vertical soil nutrient profiles include exudation from and uptake into root biomass, as well as mixing by biota that may smooth the profiles^57^. Vertical and lateral leaching of floodplain soils through the movement of groundwater may also redistribute nutrients, via advective transport of dissolved substances^54^ and later uptake into solid phases at redox interfaces and through root uptake.

Overall, the net result of organic matter deposition, redox-dependent processes, biotic uptake and mineralization is expected to be a profile of downwards decreasing concentrations of nutrients and carbon, towards stable background values ([P_const_], [N_const_], [C_const_]) at depth *Z*_*const*_. A background pool of poorly reactive P, N and C fractions is expected to be present, that may be increased during sediment deposition and lost during erosion. As the insert in Fig. 2 indicates, *Z*_*const*_ migrates vertically due to net deposition (scenario 1) or erosion (scenario 2) but is always positive as *Z* increases with increasing depth from the sediment surface. For scenarios 1 and 2, the mean annual areal retention of P during *T* years after channel construction, *m*_*P*_, is thus obtained from:

1 $$\:{m}_{P}=\left(\sum\:_{0}^{{Z}_{const}}{P}_{T}-\rho\:\left[{P}_{const}\right]\left({Z}_{const}-\varDelta\:Z\right)\right)/T$$

where the subscript *T* denotes the quantities determined *T* years after channel construction and $\:\sum\:_{0}^{{Z}_{const}}{P}_{T}$is the cumulative areal mass of P between *Z* = 0 (sediment surface) and Z_*const*_. The second term corrects for the net deposition (*ΔZ* > 0, scenario 1) or net erosion (*ΔZ* < 0, scenario 2), expressed as $\:\rho\:\left[{P}_{const}\right]\varDelta\:Z$, so that the retained mass is lower than $\:\sum\:_{0}^{{Z}_{const}}{P}_{T}$ when $\:0<\varDelta\:Z<{Z}_{const}$ or $\:\varDelta\:Z<0$, and higher when $\:\varDelta\:Z>{Z}_{const}$. As [P_const_], [N_const_] and [C_const_] are constant with time, they can be derived from cores collected at any time point between *t* = 0 and *t* = T.

**Scenarios 3 and 4** describe non-excavated channel parts in which biogeochemical equilibrium exists at the soil surface between *t =* 0 and *t =* T. The same chemical, biological and physical processes take place as for scenarios 1 and 2. As for Scenarios 1 and 2, *Z*_*const*_ migrates vertically due to net deposition (scenario 3) or net erosion (scenario 4), leading to approximately constant concentration profiles above *Z*_*const*_ between *t =* 0 and *t =* T. It follows that the only change in net P retention between *t =* 0 and *t =* T is that caused by net sediment deposition or erosion, with *m*_*P*_ obtained from:

2 $$\:{m}_{P}=\rho\:\left[{P}_{const}\right]\varDelta\:Z/T$$

**Scenarios 5 and 6** apply to areas where biogeochemical processes in the topsoil have not reached equilibrium e.g. due to limited time since excavation, high rates of erosion or deposition or limited oxygen penetration into the sediment, or where the bulk density and grain size distributions show notable vertical variations. These are typical conditions for main channel bed sediments, which are exposed to the largest variation in flow conditions and to permanent saturation. MC sediments also display the lowest elevation and thus potential exposure of older layers. Such conditions may prevent defining unambiguous [P_const_], [N_const_], [C_const_] and *Z*_*const*_. Thus, cumulative masses representing *t =* 0 and *t =* T are required down to the layers where biogeochemical retention or release may have occurred between *t* = 0 and *t* = T, referred to as *Z*_*ref*_. For scenarios 5 and 6, *m*_*P*_ is obtained from:

3 $$\:{m}_{P}=\left(\sum\:_{0}^{{Z}_{ref,T}}{P}_{T}-\sum\:_{0}^{{Z}_{ref,0}}{P}_{0}\right)/T$$

where the subscript 0 denotes the quantities determined at time *t* = 0, and $\:{\text{Z}}_{ref,0}=\underset{}{\text{max}}\{{Z}_{const,0}\:;\left|\varDelta\:Z-{Z}_{const,T}\right|\}\:$and $\:{\text{Z}}_{ref,T}$_=_$\:\underset{}{\text{max}}\left\{\left|\varDelta\:Z+{Z}_{const,0}\right|;\:{Z}_{const,T}\right\}\:$, where *Z*_*const*_ is considered as the average over multiple locations.

At the study site, we applied the framework by considering a 9-year period from *t* = 0 to *t* = 9 years. *ΔZ* was obtained from the cross-sectional surveys while the sediment core data were used to derive the values of the nutrient retention parameters. Scenarios 1 and 2 were used for the excavated floodplain and its bank, scenarios 3 and 4 for the non-excavated main channel banks below and above the floodplain level, and scenarios 5 and 6 were applied for the permanently saturated main channel bed (Fig. 1). The core collected in 2009 was assumed to represent the initial conditions in the MC at *t* = 0, and the core collected in MC at *t* = 2 years was combined with the 2009 core by vertically shifting the data by the known *ΔZ* between *t* = 0 and 2 yrs to decrease the uncertainties by increasing the number of observations. Before applying the framework, we test the assumption of biogeochemical equilibrium through evaluation of the stability of *Z*_*const*_, [P_const_], [N_const_], and [C_const_] across the core locations.

[P_const_], [N_const_], [C_const_], and *Z*_*const*_ were derived assuming cores from MC bank above FP level to be representative of all bank areas above the FP level, floodplain cores to be representative of floodplain and banks below the FP level, and the main channel bed cores to be representative of the permanently inundated MC bed. To obtain$\:\:\sum\:_{0}^{{Z}_{const,T}}{P}_{T}$, $\:\sum\:_{0}^{{Z}_{const,T}}{N}_{T}$ and$\:\:\sum\:_{0}^{{Z}_{const,T}}{C}_{T}$, we established regression equations between the cumulative masses of P, N, and C and the depth from the sediment surface for the different channel parts. The cumulative mass was obtained by summing the areal masses of the overlying layers, with the areal mass of each layer derived as $\:{P}_{T,i}={h}_{i}{\rho\:}_{i}\left[{P}_{i}\right]$ where *h*_*i*_, *ρ*_*i*_, and [*P*_*i*_] are the thickness, dry bulk density and P concentration of each slice. We selected linear regression equations without intercept in the form of $\:\sum\:_{0}^{Z}{P}_{T}={k}_{P}Z$, $\:\sum\:_{0}^{Z}{N}_{T}={k}_{N}Z$ and$\:\:\sum\:_{0}^{Z}{C}_{T}={k}_{C}Z$, where *k*_*P*,_*k*_*N*_ and *k*_*C*_ are the respective average volumetric P/N/C contents in the sediment, respectively. The tested second or third order polynomial regressions had an overall poorer fit. For scenarios 1‒4, we used *ρ* above *Z*_*const*_ determined at *t*=9 years. The mean cross-sectional net retention was obtained as a channel-part-length-weighted average. The total sedimentary retention of P, N, and C in the two-stage channel was obtained by multiplying the mean cross-sectional net retention by the reach length (820 m). The retention efficiencies (*R*_*eff*_) were computed as the retained masses in relation to the total transported loads entering the TSC in the 9-year period.

## Results

### Net deposition and erosion in the two-stage channel

Table 1 shows the mean net deposition (Δ*Z*) in different parts of the two-stage channel. During both *t* = 0‒2 years and *t* = 2‒9 years, the excavated floodplain and floodplain bank underwent net deposition while the main channel‒floodplain bank, main channel bed and main channel bank below floodplain level experienced net erosion. The SS retention efficiency of the 820 m long two-stage channel was slightly positive in the first two years after the TSC construction, but there was net release of SS during the following 7 years (Table 1). The differences in the total mass balance were largely controlled by the efficiency of the floodplain in retaining the total transported SS loads (*R*_*eff*_), which amounted up to 11% in *t* = 0‒2 years. However, *R*_*eff*_ of the floodplain was 77% lower during *t* = 2‒9 years with ~ 30% lower floodplain discharge *Q*_*FP*_ (Table 1). Other substantial differences between the two periods were the 62% (1 cm/a) higher erosion rate in the main channel bed following the floodplain construction (*t* = 0‒2 years) compared to *t* = 2‒9 years, and the 370% (0.7 cm/a) increase in the erosion rate on the MC bank below floodplain level from *t* = 0‒2 years to *t* = 2‒9 years.

**Table 1:** Mean net deposition (*ΔZ*) and SS retention efficiency (*R*_*eff*_) of the different parts of the 820 m long two-stage channel during periods *t* = 0‒2 yrs, *t* = 2‒9 yrs, and the entire 9-year period (standard deviation of the 6 cross-sections in parentheses). Negative values denote net erosion. The 9-year average values do not correspond exactly to the temporal average over the two periods because of differences in the cross-sectional geometry in different yeas. FP refers to floodplain, MC to main channel. Detailed floodplain flow properties are given in supplementary text S2 and table S2 online.

Channel part	*ΔZ* in t = 0‒2 yrs (higher Q_FP_) (cm/a)	*ΔZ* in t = 2‒9 yrs (lower Q_FP_) (cm/a)	*ΔZ* at t = 0‒9 yrs (cm/a)	*R*_*eff*_ at t = 0‒2 yrs (%)	*R*_*eff*_ at t = 2‒9 yrs (%)	*R*_*eff*_ at t = 0‒9 yrs (%)
Floodplain bank	0.1 (0.5)	0.1 (0.1)	0.1 (0.2)	0.5	1.0	0.9
Floodplain	0.7 (0.5)	0.1 (0.1)	0.3 (0.1)	11.0	2.5	4.7
MC‒FP bank	−0.8 (0.6)	−0.4 (0.9)	−0.3 (0.6)	−2.9	−1.1	−0.9
Main channel bed	−2.6 (2.0)	−1.6 (1.1)	−1.7 (0.7)	−9.3	−6.7	−7.2
MC bank below FP level	−0.2 (3.5)	−0.9 (0.9)	−0.5 (1.1)	1.2	−2.2	−1.0
MC bank above FP level	0.7 (1.7)	−1.3 (0.5)	−0.8 (0.4)	1.2	−8.5	−5.1
Cross-sectional average	0.0 (0.4)	−0.2 (0.2)	−0.2 (0.2)	0.2	−10.3	−8.6
Cross-sectional average excluding large erosion on steep banks	0.0 (0.1)	−0.1 (0.1)	−0.1 (0.1)	3.4	−6.6	−4.8

**Fig. 3:**
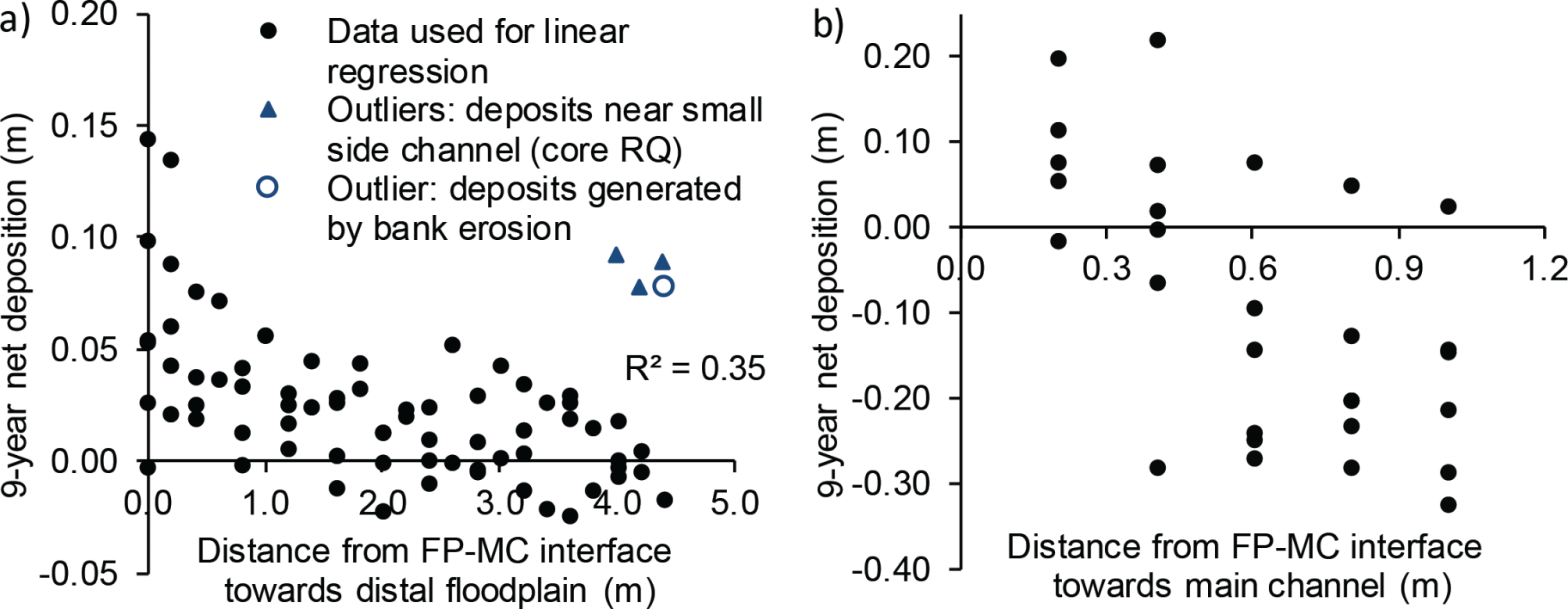
Spatial variation in the 9-year net deposition *ΔZ* as a function of distance from the FP‒MC interface (**a**) outwards towards the distal floodplain, and (**b**) inwards towards the main (low-flow) channel. Outliers are excluded from the trendline. Note that the scales of the y axes differ for visual clarity. Data from 6 cross-sections are combined in the figure.

Net deposition on the floodplain notably decreased with increasing distance from the floodplain‒main channel interface, decreasing on average to 0 at a distance of 3.7 m from the interface (Fig. 3a). As an exception, substantial floodplain deposition far from the main channel was recorded only where the adjacent floodplain bank suffered from bank erosion and downstream from a small side channel discharging to the toe of the floodplain bank, assumed to enhance local sediment supply (see Supplementary text S6 online). Floodplain deposition increased from the upstream (0.27 cm/a) to mid-stream (0.30 cm/a) to downstream (0.35 cm/a), potentially reflecting the slightly higher floodplain water depths in the downstream part (see Supplementary text S2 online). The main channel‒floodplain bank exhibited rather consistent net deposition near the FP‒MC interface, which changed to net erosion at ~ 0.5 m distance from the interface (Fig. 3b). Via this mechanism, the floodplain-like area was widened compared to the original TSC geometry of *t* = 0 yrs.

### Validation and parameterization of the framework for estimating the net sedimentary retention of P, N and C after two-stage channel construction

**Fig. 4:**
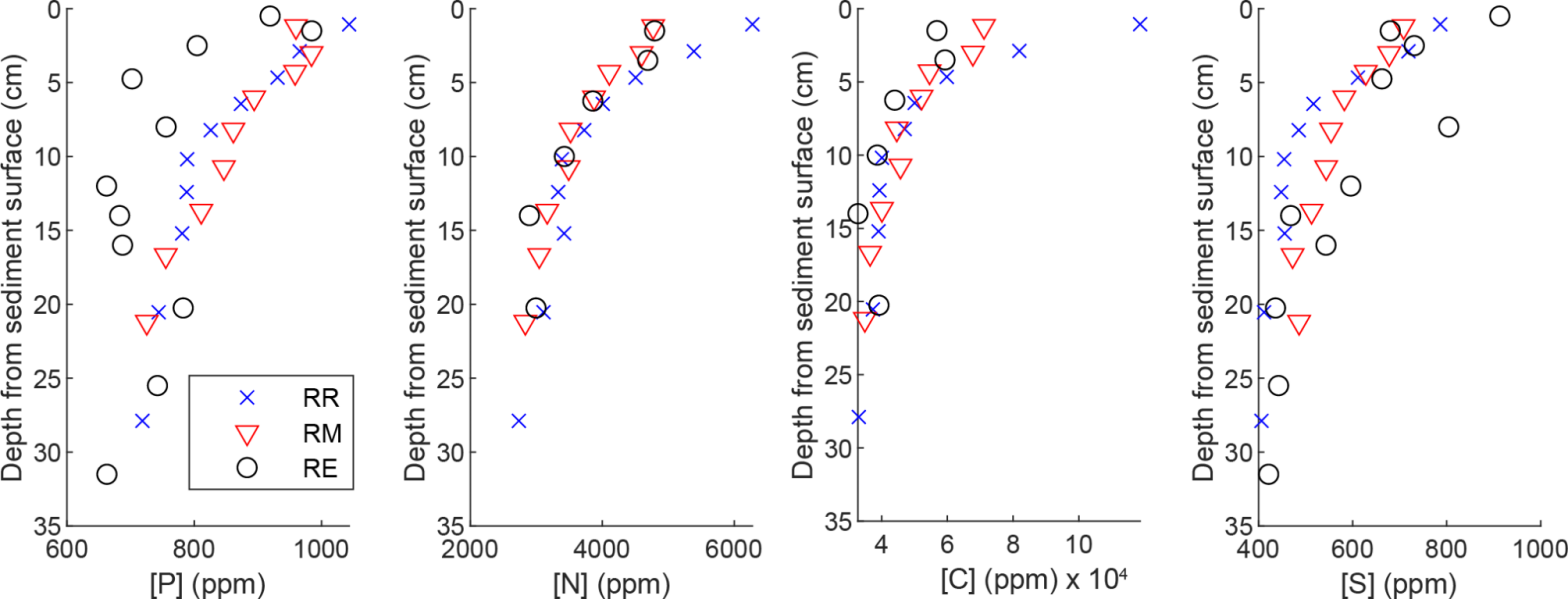
Vertical concentration distributions of phosphorus, nitrogen, carbon and sulphur in the bank sediment based on three cores. RE was sampled in November while RR and RM were collected in early summer.

**Fig. 5:**
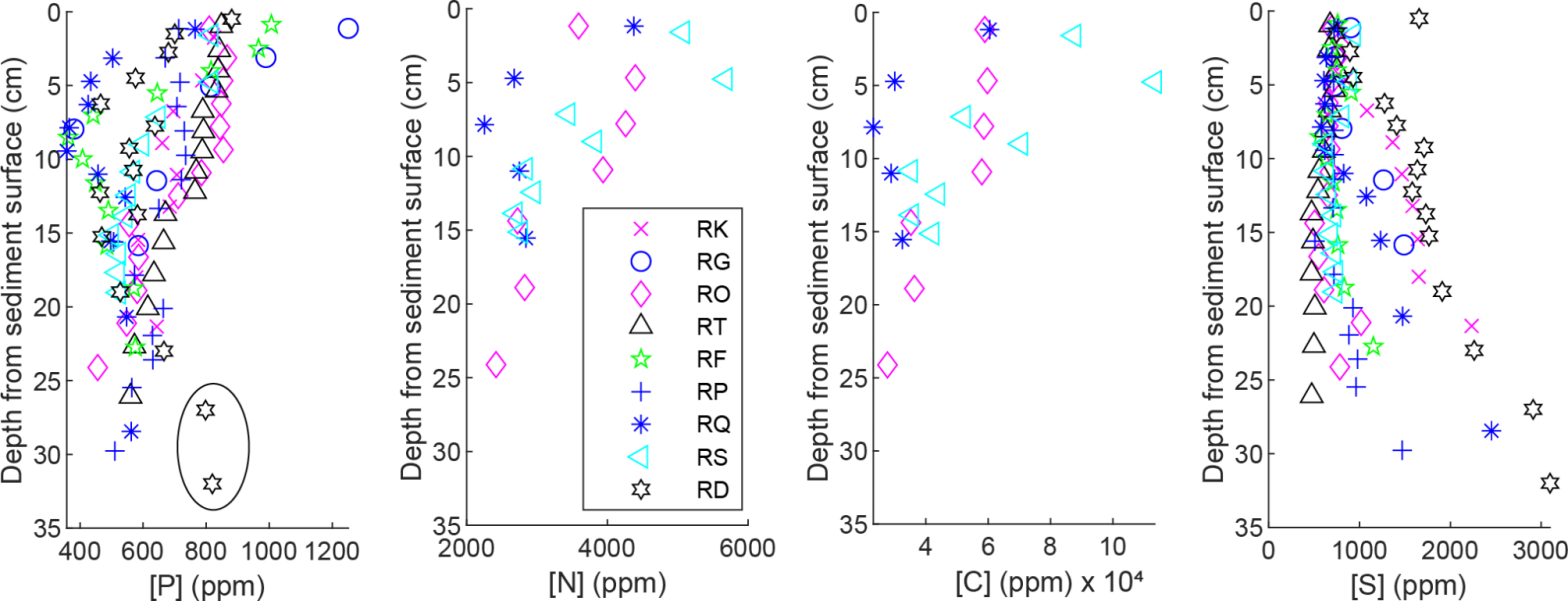
Vertical concentration distributions of phosphorus, nitrogen, carbon and sulphur in the floodplain sediment based on nine cores. Two high values of [P] below *Z*_*const*_ associated with high S encircled. The deviating slices (cores RD and RQ) explained in Supplementary text S6 online.

First, we confirm the approximate equilibrium in biogeochemical retention of nutrients assumed for scenarios 1‒4 of the framework, by analyzing the independence of *Z*_*const*_, [P_const_], [N_const_] and [C_const_] from *ΔZ*, and by assessing biomass and species richness in the vegetated TSC. For both the excavated floodplain and bank as well as for the non-excavated bank, we observed surface enrichments of P, N and C and a concave decline to background values of each element over the upper decimeters of the soil column as described by scenarios 1‒4 (Figs. 4 and 5). Below *Z*_*const*_=15 cm, most of the cores showed approximately similar, constant concentrations: [P_const_], [N_const_] and [C_const_], respectively. There were few minor deviations from the idealized profiles in cores RQ and RD, which are explained by location-specific peculiarities (Supplementary text S6 online).

**Fig. 6:**
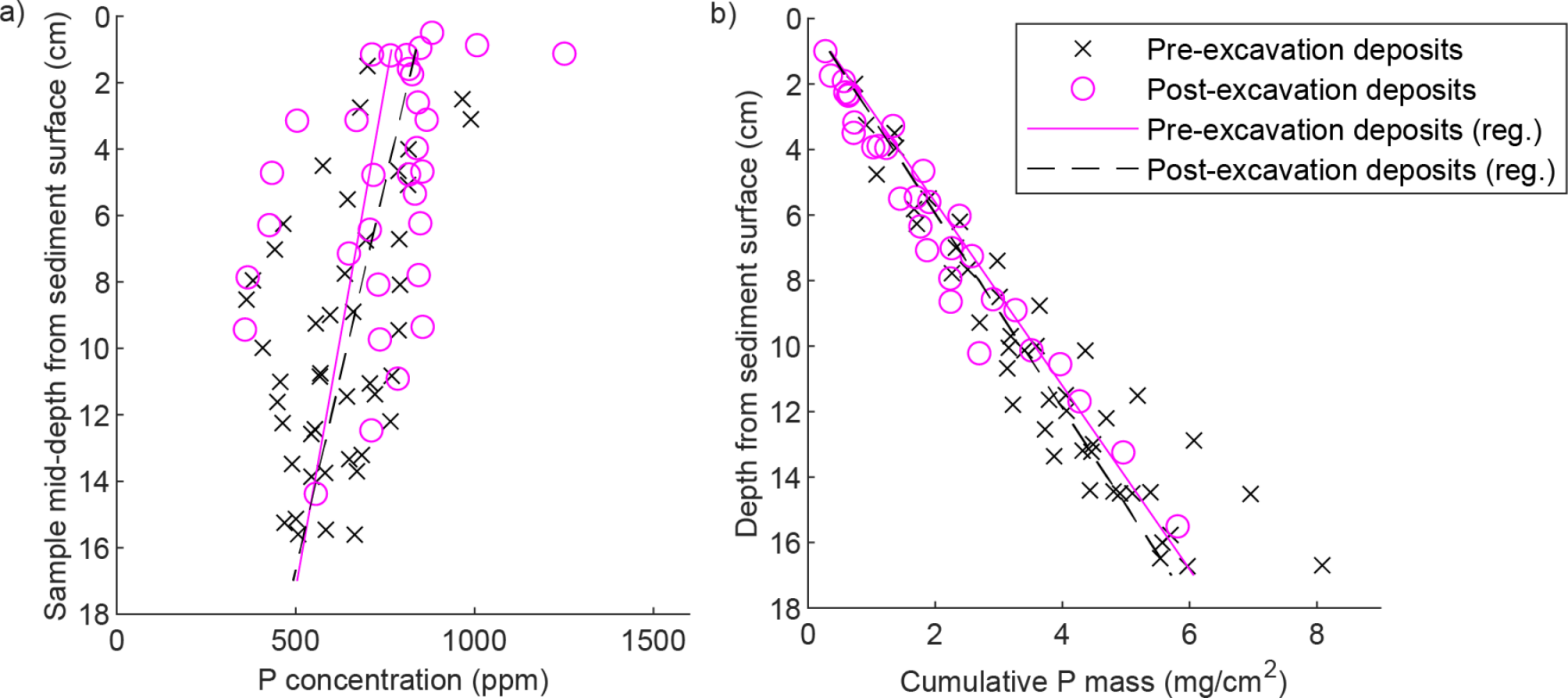
Concentrations and cumulative masses of phosphorus on the floodplain and associated linear regressions. P concentration as a function of depth from sediment surface (**a**), and cumulative P mass in the floodplain soil above a given depth from sediment surface (**b**).

Overall, *Z*_*const*_ was quite independent of *ΔZ*, following scenarios 1‒4. Consequently, chemical profiles of a given channel part are similar regardless of the degree of deposition or erosion (see e.g. P profiles from bank cores RM and RR, with 6 cm and 12 cm of erosion, respectively, Fig. 4). Further, the average concentrations and cumulative mass of nutrients as a function of depth from the soil surface are comparable for pre-excavation and post-excavation soil horizons, when all depths for all sediment cores are considered together (see example for P for floodplain cores, Fig. 6). The similarity of the vertical profiles for the FP cores, which cover a range of stages of vegetation development (Supplementary Fig. S5 online), suggests that *Z*_*const*_, [P_const_], [N_const_] and [C_const_] become quickly established within the 9-year timeframe of the study and remain similar despite ongoing changes in vegetation. We note that areal biomasses of the dominant natural grassy vegetation in the TSC were already at *t*= 2 years^9^comparable to those of a slightly lower latitude natural floodplain^58^, implying rapid establishment of topsoil conditions influenced by biogeochemical cycling. Furthermore, the excavated FP and FP banks harbored 82 plant species at *t*= 10 years^16^, which is notably higher than for any of 13 streams restored 5‒25 years ago at a slightly higher latitude^59^ and also supporting the rapid establishment of biogeochemical equilibrium. Having demonstrated how the assumptions of the framework hold for the study site, we report the values of the parameters for each channel part in Table 2 while the cumulative mass distributions of $\:\sum\:_{0}^{Z}{P}_{T}$, $\:\sum\:_{0}^{Z}{N}_{T}$ and$\:\:\sum\:_{0}^{Z}{C}_{T}$ are shown for the floodplain and bank in Supplementary Fig. S6 online.

**Table 2:** Values of the parameters for the three sampled channel parts.

Channel part	Scenario	*Z*_*const,0*_ (cm)	*Z*_*const, T*_ (cm)	[*P*_const_] (ppm)	[*N*_const_] (ppm)	[C_const_] (ppm)	*k*_*P*_ (mg *P*/cm^3^)	*k*_*N*_ (mg *N*/cm^3^)	*k*_*C*_ (mg C/cm^3^)
Floodplain	1‒2	0	15	579	2710	34,600	0.0004	0.0018	0.0266
MC bank above FP level	3‒4	15	15	720	3020	36,700	0.0005	0.0025	0.0334
Main channel bed	5‒6	17	5	NA	NA	NA	0.0004 at *t* = 0 yrs, 0.0004 at *t* = 9 yrs	0.0019 at *t* = 0 yrs, 0.0017 at *t* = 9 yrs	0.0260 at *t* = 0 yrs, 0.0201 at *t* = 9 yrs

**Fig. 7:**
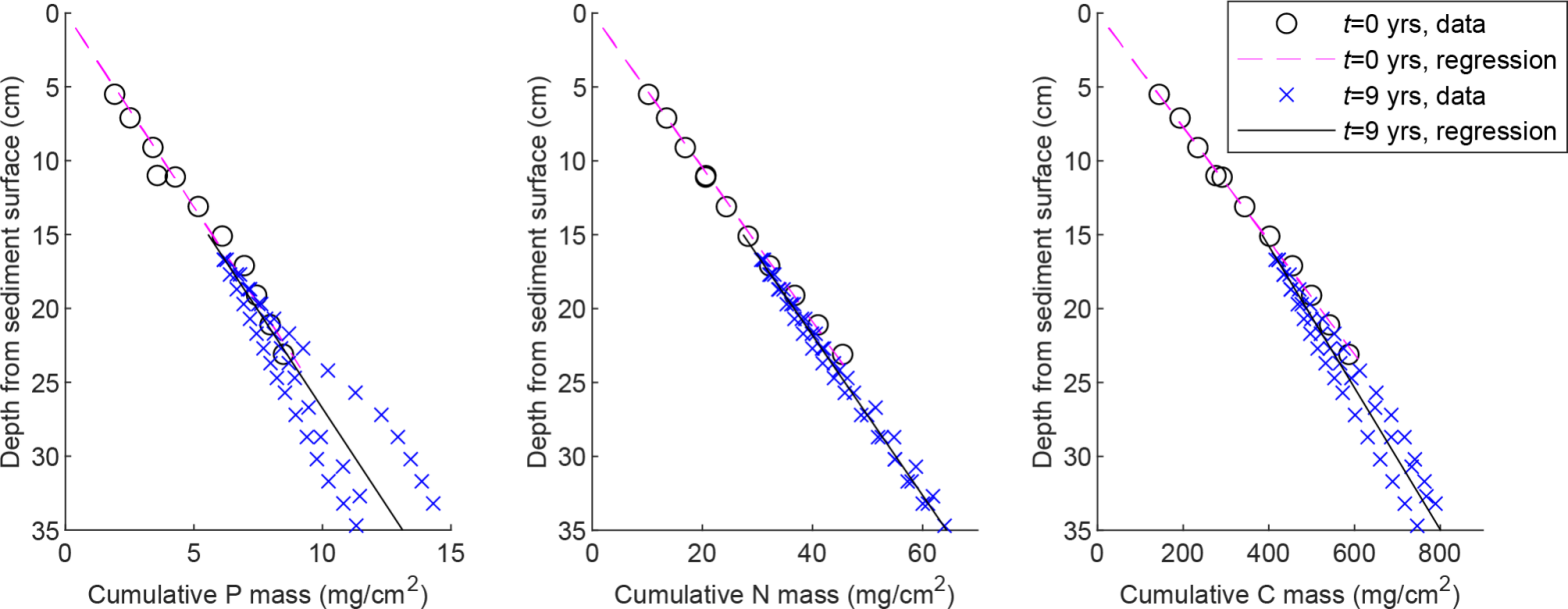
Cumulative masses of phosphorus (P), nitrogen (N), and carbon (C) in the main channel bed. The origin of the y axis is located at the sediment surface as measured at *t* = 0 yrs.

For the main channel bed, the vertical concentration distributions (Supplementary Fig. S7 online) differed between the cores as expected for scenarios 5‒6. To compute the cumulative masses above *Z*_*ref*,_, we used values of *Z*_*ref,0*_ =21 cm and *Z*_*ref, T*_=5 cm as derived based on the average retention parameters of *ΔZ* =−16 cm (Table 1), *Z*_*const,0*_ =17 cm, and *Z*_*const,9*_ ~5 cm (Supplementary Fig. S7 online; Table 2). The values of *k*_*P*_ and *k*_*N*_ as well as the cumulative masses in the overlapping MC bed sediment layers, i.e. approximately at the depth of 16–23 cm from the sediment surface at *t* = 0, were roughly similar between *t* = 0 and *t* = 9 years for P and N (Fig. 7; Table 2), which would indicate no biogeochemical retention/release for vertically uniform sediment bulk density. However, biogeochemical retention/release cannot be ruled out since it is likely masked by the differences in the bulk densities and grain size distributions of the different MC bed sediment layers. For C, cumulative masses in the overlapping layers and *k*_*C*_ were 20% and 23% lower, respectively, at *t* = 9 compared to *t* = 0 years, indicating losses likely attributable to the partial oxidation of the C-rich Littorina sea deposits (Supplementary text S6 and Fig. S7 online).

### Net retention and release of SS, phosphorus, nitrogen and carbon in different parts of the channel

Table 3 shows the averaged areal net retention of suspended sediment and sedimentary phosphorus, nitrogen, and carbon in the different parts of the TSC over the 9-year period. The main channel bed and banks experienced on average notable net release whereas the floodplain and floodplain bank were regions of net retention. The reach-scale variability was reflected in the coefficient of variation (*c*_*v*_, standard deviation divided by the absolute value of the mean), which was largest for both the MC‒FP bank and the MC bank below FP level (*c*_*v*_=1.9 for the 4 substances). The most consistent results were obtained for the floodplain and the main channel bed (*c*_*v*_=0.12‒0.41). The cross-sectionally averaged net retention was negative for SS, P and N and slightly positive for C (Table 3). This difference reflects the stronger relative surface enrichment in C than in P and N in areas of positive net retention such as floodplain and floodplain bank (Fig. 4).

**Table 3:** Annually averaged net retention of SS, and sedimentary retention of phosphorus, nitrogen and carbon in different parts of the two-stage channel over the 9-year period (standard deviation of the 6 cross-sections in parentheses). FP refers to floodplain, MC to main channel. Negative values denote net release. 2 significant digits.

Channel part	SS (kg/m^2^/a)	P (g/m^2^/a)	N (g/m^2^/a)	C (g/m^2^/a)
Floodplain bank	0.56 (0.73)	2.0 (0.69)	14 (2.9)	200 (35)
Floodplain	1.3 (0.5)	1.3 (0.36)	8.9 (1.7)	170 (22)
Main channel‒floodplain bank	−1.5 (2.9)	−1.0 (2.0)	−4.9 (9.4)	−62 (120)
Main channel bed	−11.6 (4.7)	−6.3 (1.6)	−32 (7.8)	−470 (90)
MC bank below floodplain level	−2.6 (5.0)	−1.8 (3.5)	−8.4 (16)	−110 (210)
MC bank above floodplain level	−3.6 (1.6)	−3.4 (1.5)	−14 (6.3)	−180 (76)
Cross-sectional average	−0.97 (0.72)	−0.45 (0.50)	−0.98 (2.2)	24 (28)

**Fig. 8:**
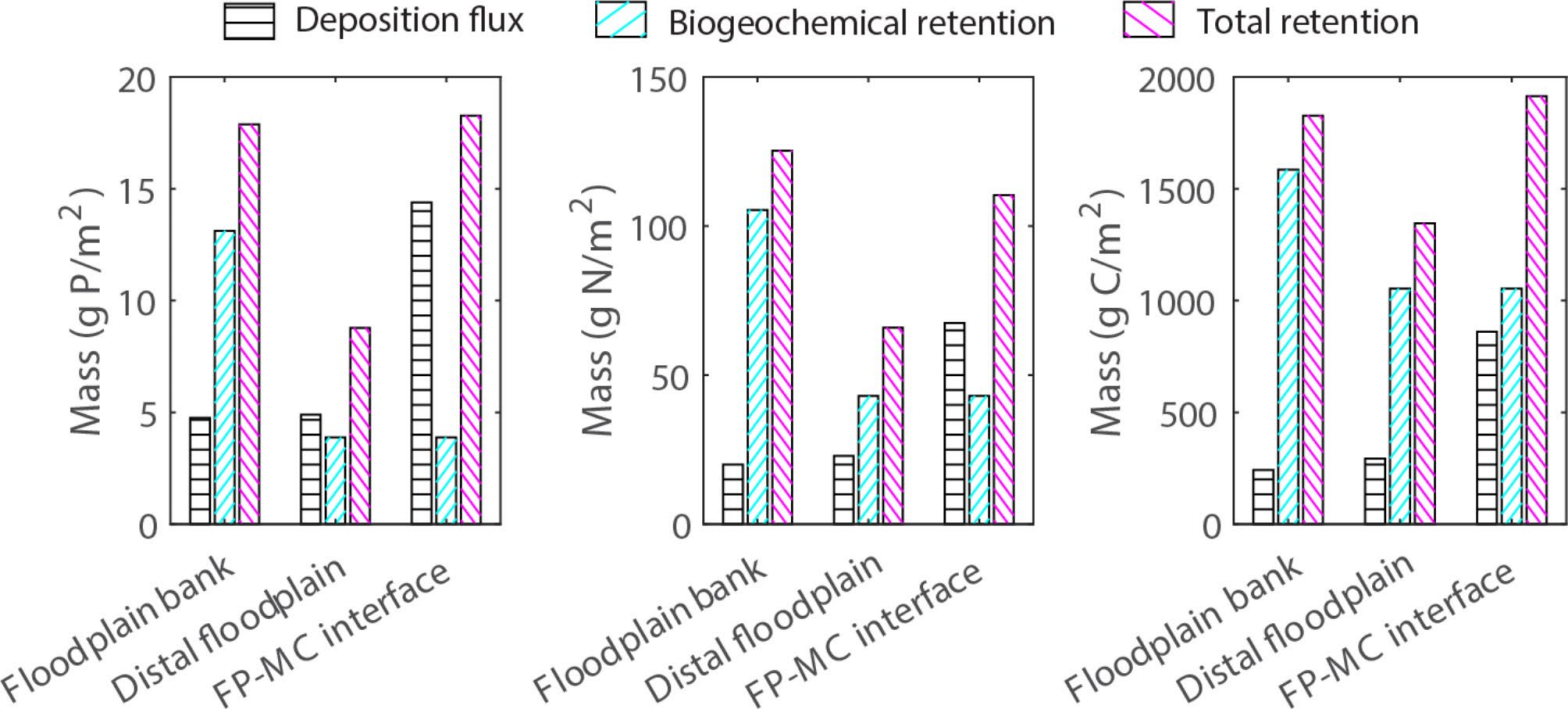
9-year deposition, biogeochemical retention and total retention of P, N and C on newly exposed soil surfaces.

Figure 8 shows the fraction of sedimentary nutrient retention attributed to physical deposition and biogeochemical processes, respectively, for the newly exposed (post-excavation) surfaces over the 9-year period. For this analysis, the floodplain was further sub-divided into a ~ 1.5 m wide FP‒MC interface area and a 3 m wide distal floodplain (Fig. 1) to consider the decrease in floodplain SS deposition with increasing distance from the main channel (Fig. 3a). The deposition fluxes constituted a larger share of the net sedimentary retention for P followed by N compared to C. The deposition fluxes exceeded or equaled the biogeochemical retention only at the FP‒MC interface, which showed the highest deposition in the system, and for P on the distal floodplain. The biogeochemical retention on the floodplain bank was 2.5‒3.4 times that on the more frequently inundated distal floodplain for P and N.

**Fig. 9:**
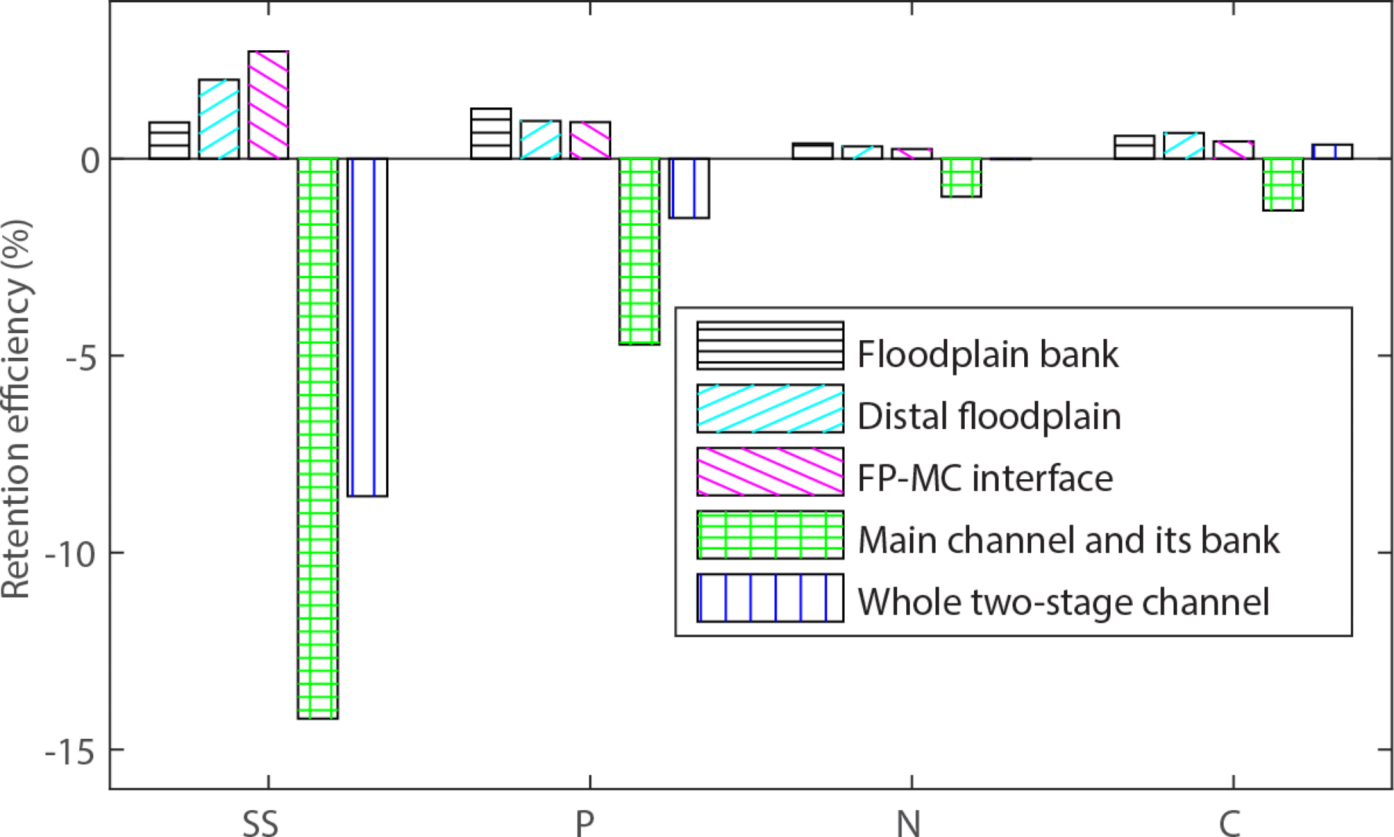
Retention efficiencies of the different channel parts of the 820 m long two-stage channel (TSC) during the 9-year period, and the total retention percentage of the whole TSC. The total efficiency was 0.0% for N.

The sedimentary retention efficiencies of the newly constructed TSC parts, the non-excavated main channel and the whole TSC are shown in Fig. 9, derived from the total computed retention along the 820 m TSC and the transported loads during the 9-year period. For each channel part, the total transported load for the entire cross-section was used in the calculation, such that the efficiencies can be summed to a cumulative total. The 1.5 m wide FP‒MC interface region generated a similar total net retention to that of the 3 m wide distal floodplain for SS and P, respectively, while the distal floodplain was more important for C and N. Summed *R*_*eff*_ of the floodplain and floodplain bank was 5.7% for SS and 3.2% for P (with the 9-year averaged total loads of 9640 kg/km^2^ catchment area/a for SS and 24.9 kg/km^2^ catchment area/a for P). The corresponding *R*_*eff*_ was lower for N (0.95%, with the total load of 571 kg/km^2^ catchment area/a) and C (1.7%, with the total load of 5560 kg/km^2^ catchment area/a). *R*_*eff*_ of the whole TSC was however negative, due to the large losses of sedimentary material from the main channel bed and banks.

## Discussion

### Overall applicability of the sedimentary nutrient retention framework

Our study improves understanding on the functioning of two-stage channels, which are a relatively new and still sparsely studied nature-based solution for flood and drainage water management in agricultural streams^11,16,17^. The proposed and validated framework considers the medium-term net balance between retention and release of carbon, nitrogen and phosphorus in the sediments and soils of the channel cross-section. Sediments are a particularly important sink and source for P, for which experimentally obtained mass balances in TSCs are scarce^26^. We are not aware of other studies evaluating the nutrient retention efficiency of TSCs covering the time period from floodplain construction to well-established biogeochemical conditions.

The applied experimental methods, including the sediment cores and estimates of the transported loads at daily time steps, improve the reliability of the retention efficiency evaluation compared to the typically used snapshot water quality sampling^33^. Our methods take into account the processes during the entire 9-year hydrograph and the changes in the sediment budget caused by non-equilibrium bed load transport, which are not reliably captured by most other studies because the commonly used grab water sampling easily misses the highest loads and coarser fractions^8,33,35,37^. In contrast to studies on short-term depositional fluxes^40,60^, our results implicitly take into account the potential erosion during the rising limb of the next flood season^61^and the fact that the concentrations in the deposited sediment may be lowered by mineralization of plant remains and desorption in response to reducing conditions created by sediment burial^62^.

The developed framework is applicable at other TSC sites on fine-grained, low-gradient soils. Most of the parameter values need to be determined based on site-specific sampling, but the framework helps in prioritizing the required sampling and interpretation of the results. For instance, it would be sufficient to collect sediment cores at *t* = 0 in the channel parts assumed to have non-equilibrium biogeochemical conditions (scenarios 5 and 6), i.e., in most cases the main channel bed or areas of substantial erosion or deposition (*ΔZ* > ~ 2–3 cm/a), and only at the end of the study period on all key channel parts (floodplain, bank and MC bed). Further, sedimentary nutrient analyses are required only above *Z*_*const*_ for scenarios 1‒4, i.e., for the floodplain and channel banks in most cases. The channel design, such as the floodplain elevation and thus inundation frequency, FP width, and whether the floodplain is located on one or both sides of the main channel, are expected to influence the values of *ΔZ*^27,30^ and *Z*_*const*_^63,64^. The values of [P_const_], [N_const_], and [C_const_] are mainly affected by the properties of the parent soil. Accepting some uncertainty, the obtained values of some parameters, including *Z*_*const*_, are likely transferable between sites with roughly similar soils, hydro-climatological conditions, and floodplain inundation frequencies controlling the multi-annual-scale biogeochemical retention.

When applying the framework, we note that besides the deposition and biogeochemical retention of waterborne nutrients, possible additional sources of N and P in TSC sediments include direct deposition of atmospheric particulate N and P and local fixation of atmospheric N_2_ by diazotrophic organisms^65^. However, at our TSC case site, we expect that these sources are negligible. The estimated atmospheric deposition (~ 0.4 g N/m^2^/a and 0.015 g P/m^2^/a in Southern Finland^66,67^) comprised less than 5% and ~ 1% of the N and P retention, respectively. Furthermore, the abundance of plant species associated with strong N_2_-fixing functionality, such as *Alnus*, is low in the TSC area, therefore direct fixation of atmospheric nitrogen is expected to be minor.

### Physical performance of the investigated two-stage channel

The two-stage channel functioned according to expectations in that the floodplain significantly retained nutrients (Table 3) while the low-flow channel did not suffer from siltation (Table 1; Fig. 1). The TSC design thus ensured that the drainage depth did not decrease with time, contributing to maintaining the conveyance capacity. Although there are no data of the channel geometry soon after the last conventional dredging, the depth of a S-rich marker horizon in the *t*= 0 core (~ 17 cm) indicated that the conventionally dredged channel had undergone notable siltation prior to TSC construction (see details in Supplementary text S6 and Figure S7 online). The flushing of the fine bed deposits accumulated after the last conventional dredging supported the hypothesized enhanced self-cleansing capacity of the TSC design compared to trapezoidal cross-sections with wide beds^20,21^, but was in contrast to several Swedish sites with notable MC bed deposition^27^. An advantage of the TSC design is that the geotechnical adjustments on the excavated floodplain banks, occurring mainly in the first few years when vegetation is not fully established (Table 1), do not block the low-flow channel. Via these mechanisms, an optimal TSC design likely reduces the need for ecologically harmful re-dredging, which may improve conditions for stream biota^18,23^. TSCs realized with good self-cleansing capacity and more natural-like flow conditions in the low-flow channel enable restoration of aquatic habitats while the improved floodplain connectivity likely enhances riparian species diversity^16,49^.

At our site, the decreasing erosion rate with time (Table 1) suggests that the loss of material from the channel bed will substantially diminish in the second decade after the floodplain construction. Most of the loose deposits have been flushed away in the first decade, as shown by the sulfur-rich marker layer coming ~ 12 cm closer to the sediment surface in later cores (Supplementary text S6 and Figure S7 online). Further field confirmation is warranted to determine whether the TSC design that presumably leads to higher flow velocities in the low-flow channel compared to the conventional re-dredging causes unacceptable erosion of the original channel materials under various sedimentological contexts.

### Implications on the design and maintenance of two-stage channels

At the investigated study site, biogeochemical retention constituted an important share of the total retention on the excavated surfaces for all substances considering a 9-year time frame (Fig. 8). However, since the biogeochemical retention is expected to reach equilibrium under the mostly oxidizing prevailing conditions, the SS and sedimentary nutrient mass balance are in the long run controlled by the erosion and deposition fluxes. The correct elevation of the floodplain is one of the most important design considerations as the floodplain deposition increases with increasing inundation frequency and higher floodplain discharge (Table 1, in accordance with earlier studies^27,30^). Additionally, our site demonstrated the importance of taking into account the resuspension-prone bed deposits accumulated after the earlier conventional dredging when implementing the TSC design for reaching SS and nutrient load reductions (Table 3; Fig. 9). The design level of the floodplain can be set lower in order to achieve the desired frequency of floodplain inundation despite the increasing flow area in the MC due to resuspension from the channel bed. Alternatively, the loose main channel deposits may be removed, or the flushing can be decreased by constructing natural-like rocky sills^37,68^.

At the present site with SS and nutrients distributed to the floodplain mainly from the main channel, the distal floodplain and floodplain banks were notably less efficient in generating deposition compared to the ~ 1.5 m wide floodplain‒main channel interface (Fig. 8). This finding together with previous studies^69,70^highlights the need to understand the flow pathways to enable the optimal design and maintenance of vegetative NbS. If the sub-surface or stormwater drains discharge to the main channel and there is little surface runoff directly to the floodplain, the retention of particulate substances becomes supply-limited if the floodplain is covered by emergent grassy vegetation that typically limits the transverse mixing^71^. At the investigated site, the floodplain vegetation was emergent during most autumn high flows, when a large part of the total loading occurs, with very little additional deposition observed at floodplain width-to-depth ratios larger than ~ 6 (Fig. 3a).

To enhance the net retention, the channel design and maintenance should ensure the substances are efficiently spread on the vegetated floodplains. The 3-fold deposition rates for P, N and C on the floodplain‒main channel interface compared to the distal floodplain (Fig. 9) and the 4-fold SS deposition during the period with greater annual floodplain discharge *Q*_*FP*_ (Table 1) indicate the magnitude of potential improvements in retention. Discharging sub-surface drain flows onto the floodplain and constructing narrower floodplains on both sides of the main channel will likely improve the sediment and nutrient retention^27^. In addition, future research should estimate the potential to increase deposition by increasing the floodplain flows and mixing from the main channel e.g. through vegetation maintenance^72^and through increasing the floodplain inundation frequency by construction of e.g. low rocky sills^37,68^.

### Efficiency of the TSC design in managing SS and nutrient loads: upscaling sedimentary retention

The results revealed that the total rates of sedimentary nutrient retention vary by up to 10-fold between different cross-sectional parts of the two-stage channel (Table 3). Because of the flushing of the earlier bed deposits, the total retention efficiency of the TSC was negative for SS and P over the first 9 years (Table 1; Fig. 9), highlighting that earlier channel modifications can strongly influence the cross-sectional-scale net retention. We are aware of annual experimental SS and TP retention efficiencies for only few agricultural TSCs, at one of which very high net retentions of 11% for SS and 20% for TP were observed per 100 m of reach length in a very small catchment^26^. In another study, TP loads were reduced by ~ 20% for two-sided floodplains with reach lengths of ~ 0.3–1.7 km^27^. At our site, the floodplain P and N retention in the short study reach averaged 0.27% and 0.08% per percentage point of the agricultural channel network length converted into two-stage geometry, P retention being approximately half of the corresponding Fig. (0.5%) obtained for a US watershed through scenario modelling^32^. TSC sites have also shown high potential for SRP load reductions (4.2‒5.5% per 100 m reach length^8,26^).

The data allows us to begin evaluating how the sedimentary retention performance of the studied TSC could contribute to achieving catchment-scale nutrient load reduction targets. Herein, we considered the retention on the floodplain and floodplain banks as a net benefit of the TSC compared to conventional dredging^16^. The net sedimentary retention efficiency *R*_*eff*_ on the FP bank and FP constituted 0.69% of the total transported SS load and 0.39% of the total P load per 100 m of TSC (Fig. 9). *R*_*eff*_ for N and C was notably lower at 0.12% and 0.20%, respectively, per 100 m of TSC. Load reductions required to reach a good ecological status in the receiving inland waters and Gulf of Finland are on average 55% for P and 15.5% for N^73,74^, implying that TSCs, if employed as a major component of load reduction strategies, would need to cover large areas of their catchments to have a significant influence. In the following estimates, we scaled the nutrient inputs along the channel network taking into account the changes in the upstream catchment area and considered a constant floodplain inundation frequency such that the *R*_*eff*_ values are similar in different sections of the TSC. Using the 9-year values of nutrient retention from this study indicates that converting ¾ of the channel network to the TSC design would provide 13‒14% of the target load reductions in the study catchment. Assuming doubled *R*_*eff*_ due to enhanced spreading of nutrients to the floodplain, achieving 16‒17% of the target reductions would require converting ~ 22% of the channel network to TSCs. We note that these percentages cannot be directly generalized to other catchments due to differences in the topology of the channel network and the spatial distribution of loading along the network, but they do provide a starting point for assessing the broader potential impacts of TSCs in nutrient retention.

When estimating the overall water quality impacts of TSCs, processes not reflected in the sedimentary net retention should also be considered. While the life cycle of the grassy vegetation is implicitly considered, the uptake of nutrients into the standing stock of woody vegetation adds to the removal efficiency. In addition, the observed sedimentary retention of N, the relatively high near-surface C content of 6‒10% (Figs. 4 and 6) and the fact that sediment may become anoxic are expected to contribute to efficient N removal through denitrification at such sites where floodplains are frequently inundated during non-freezing temperatures^15,28,38^. In the future, the developed framework should be applied in settings where the TSC design can be compared to concurrent conventional re-dredging.

## Conclusion

Our 9-year experimental investigation and the developed framework have provided novel understanding of the medium-term net sediment deposition and net sedimentary retention of nutrients when a trapezoidal-shaped, conventionally dredged channel is converted into a two-stage channel (TSC). TSCs with an excavated vegetated floodplain are a relatively new nature-based solution for flood and agricultural water management from the viewpoint of combining the technical needs for drainage and flow conveyance and the ecological needs, such as enhanced water quality. We are not aware of other works where the mass balances of suspended sediment (SS) and sedimentary phosphorus (P), nitrogen (N) and carbon (C) have been evaluated starting from immediately after the excavation of the floodplain until the establishment of stable biogeochemical conditions. The validated framework is based on relatively straightforward analyses of spatially representative sediment cores and is useful for designing the required sampling and for calculating the retention efficiencies at further sites with cross-sectionally variable excavation history and biogeochemical conditions. The investigated TSC case functioned according to the expectations in that the floodplain retained SS and nutrients while the low-flow channel proved to be self-cleansing, maintaining the drainage depth, flow area and thus the conveyance capacity. Because the TSC design generated resuspension of the sediment deposited in the main channel after the last conventional re-dredging, the total sedimentary retention efficiency was negative (−8.6‒0.0%) for SS, P and N over the examined first 9 years. If the flushing of the main channel sediments is properly addressed in the design and maintenance of TSCs, the TSC design is expected to provide a net sink of SS and sedimentary nutrients over the expected channel life cycles of several decades. The framework provides estimates of the physical deposition and biogeochemical retention fluxes, and the gained understanding of the dominant processes in the characteristically different parts of the channel cross-section enables up-scaling the results to catchment scale. Finally, our findings indicate several ways in which TSCs could likely be made more efficient in reducing the downstream transport of SS and nutrients, but these require verification in further controlled investigations.

## Electronic supplementary material

Below is the link to the electronic supplementary material.

Supplementary Material 1

## Data Availability

The data used in this paper are publicly available through Zenodo (https://zenodo.org/records/14581160).
