# Supplementary material for: Evaluating multiannual sedimentary nutrient retention in agricultural two-stage channels
Source: Sci Rep. 2025 Jan 3;15:722. doi: 10.1038/s41598-024-84956-2 (PMC11698719; doi:10.1038/s41598-024-84956-2)
Supplement: Supplementary file 1 — Supplementary Material 1 [file 41598_2024_84956_MOESM1_ESM.pdf]

Supplementary information for Västilä, Kaisa[1,2,*] & Jilbert, Tom[3] 2024 Evaluating multiannual sedimentary nutrient retention in agricultural two-stage channels. Scientific Reports, doi: 10.1038/s41598-024-84956-2

[1] Primary institution: Department of Built Environment, Aalto University School of Engineering, Espoo, Finland

[2] Secondary institution: Marine and freshwater solutions unit, Finnish Environment Institute, Helsinki, Finland

[3] Environmental Geochemistry group, Department of Geosciences and Geography, University of Helsinki, Helsinki, Finland

*Corresponding author: kaisa.vastila@aalto.fi; kaisa.vastila@syke.fi

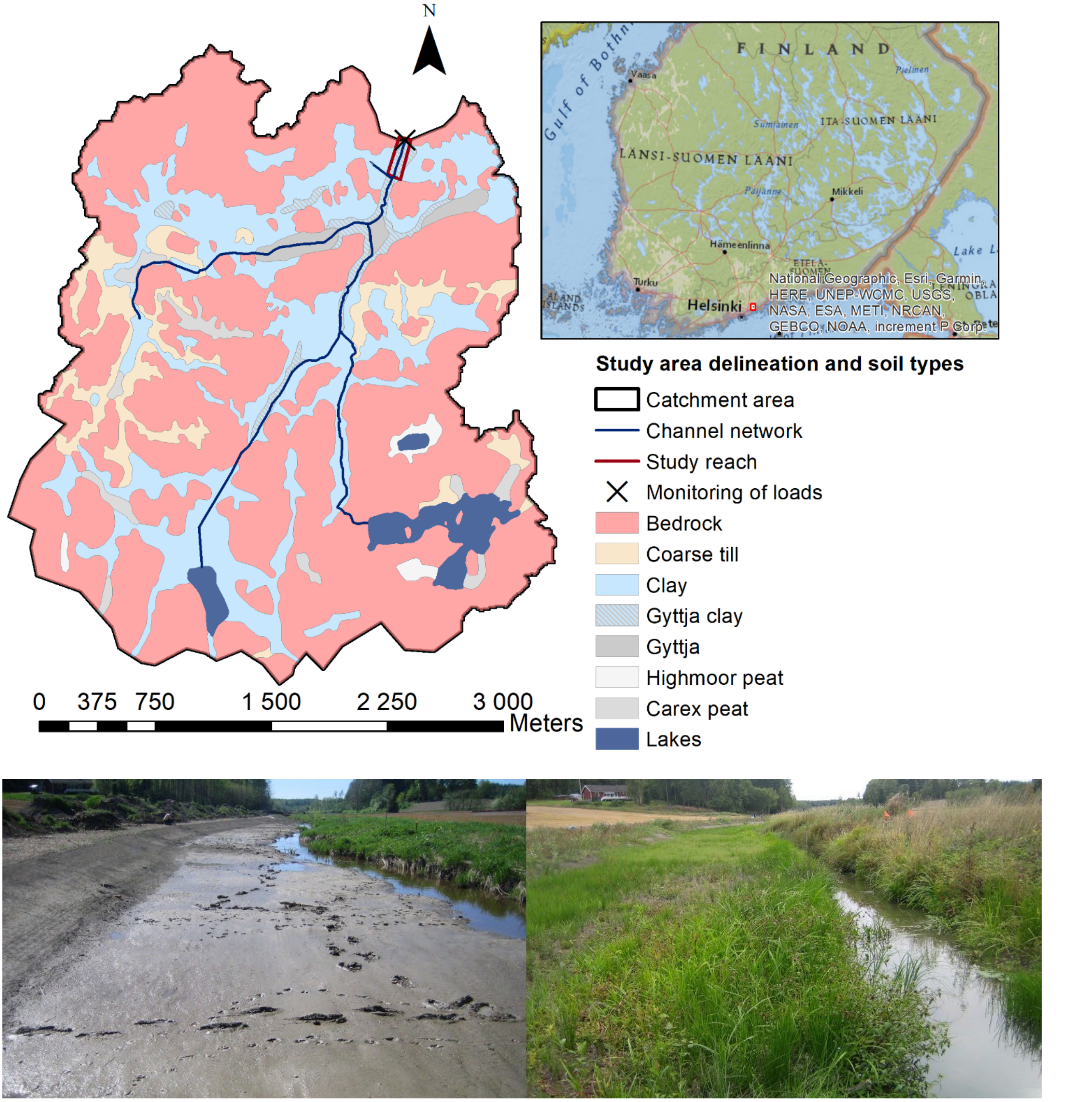

Figure S1. Delineation of the study area and soil types of the Ritobäcken catchment (top) and the two-stage channel study reach with the excavated floodplain and floodplain bank soon after the construction (bottom left) and after vegetation establishment (bottom right). The maps were created by ArcMap version 10.8 (https://www.esri.com/en-us/arcgis/products/arcgis-desktop/resources).

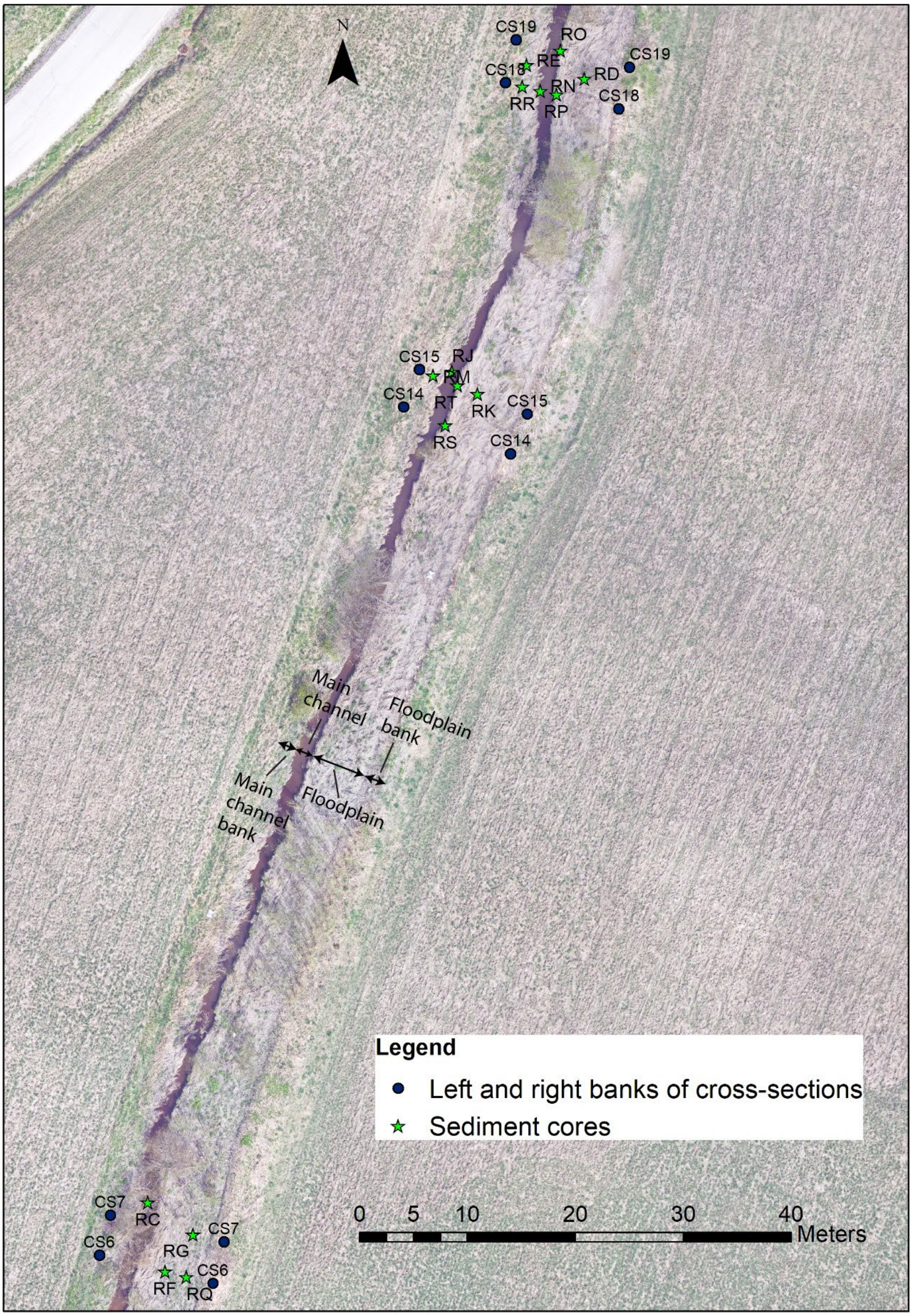

Figure S2. The study reach with the six monitored cross-sections and locations of the collected sediment cores (the accuracy of the core locations is ~0.2 m).

Text S1 Evaluation and calibration of WSFS-VEMALA for obtaining discharges and total loads

To evaluate the retention efficiency of the Ritobäcken two-stage channel, the discharges (Q) and loads of suspended sediment (Q_{SS}), total phosphorus (Q_{TP}), total nitrogen (Q_{TN}) and total organic carbon (Q_{TOC}) were estimated with the WSFS-VEMALA model[1]. WSFS-VEMALA covers ~90% of Finnish watersheds and has been widely used for operational watershed management and research purposes[2]. The model yielded output at a daily time step at the outlet of the Ritobäcken catchment located directly downstream from the two-stage channel reach ("Monitoring of loads", Figure S1). The load calculation of the WSFS-VEMALA is routinely pre-calibrated against water samples reported to the national database. At Ritobäcken, the model had been pre-calibrated based on 36–42 water samples for the suspended sediment concentration (SSC), total phosphorus concentration (TP), and total nitrogen concentration (TN), as well as 14 samples for the total organic carbon concentration (TOC). 94% of the samples were collected after year 2008, thus representing well the present study period, mainly as part of routine monitoring by different environmental authorities.

The performance of the WSFS-VEMALA was assessed against the available observations of Q, Q_{SS}, Q_{TP}, Q_{TN} and Q_{TOC}. For Q, Q_{SS}, and Q_{TP}, we used data from a continuous monitoring station located at the model output location, with Q and SSC (particles >1.2 µm) measured at 5–15 minute time steps by site-calibrated pressure and optical backscatter sensors installed downstream from a well-mixed culvert[3]. Reliable data for both discharge and SS load were available from November 2009 to April 2012, i.e. from ~4 months before to 2 years after the TSC construction. Additionally, TP was determined based on SSC since phosphorus is mainly particle-bound at this clayey site with high correlation between TP and SSC (r^2=0.97, p<0.001)[4], and as reported for similar clayey catchments[5]. For Q_{TN} and Q_{TOC}, we used the water samples from the national database collected between November 2009 and the end of the 9-year period (summer 2019) for model validation.

The performance of the WSFS-VEMALA was evaluated based on the mean absolute error (MAE), mean relative error (MRE), Nash–Sutcliffe efficiency (NSE), root mean squared error (RMSE), cumulative error (CE) and relative cumulative error (RCE, Eqs. 1–6) using the monthly values for Q, Q_{SS}, and Q_{TP} and the daily values coinciding with the dates of the collected grab water samples for Q_{TN} and Q_{TOC}. The main performance criteria was the RCE since the purpose of the modelling was to obtain the most reliable estimate of the total transported loads over the 9-year study period.

$$MAE = \frac{1}{n}\sum_{i=1}^{n}|y_i - \hat{y}_i| \quad (1)$$

$$MRE = \frac{100\%}{n}\sum_{i=1}^{n}\frac{|y_i-\hat{y}_i|}{y_i} \quad (2)$$

$$NSE = 1 - \frac{\sum_{i=1}^{n}(\hat{y}_i-y_i)^2}{\sum_{i=1}^{n}(y_i-\bar{y}_i)^2} \quad (3)$$

$$RMSE = \sqrt{\frac{1}{n}\sum_{i=1}^{n}(y_i - \hat{y}_i)^2} \quad (4)$$

$$CE = \sum_{i=1}^{n}\hat{y}_i - \sum_{i=1}^{n}y_i \quad (5)$$

$$RCE = \frac{\sum_{i=1}^{n}\hat{y}_i-\sum_{i=1}^{n}y_i}{\sum_{i=1}^{n}y_i} \quad (6)$$

where $\hat{y}_i$ and y_i denote the modelled and measured values at time i; $\bar{y}_i$ denotes the mean of measured values and n denotes the number of data points.

The pre-calibrated model provided reliable results for all variables except Q_{SS} (Table S1, Figure S3). The NSE of 0.92–0.98 and the over-estimation of the total cumulative values by -5.4%–6.7% indicated that the pre-calibrated model could acceptably describe Q_{TP}, Q_{TN} and Q_{TOC}. The performance was only slightly weaker for Q. By contrast, the pre-calibrated model performed unsatisfactorily in predicting Q_{SS}, as indicated by the negative NSE and high RMSE. Q_{SS} was systematically over-predicted during the spring snowmelt (April) and autumn high flows (Oct–Dec), resulting in a 79% over-prediction of the cumulative Q_{SS} in the 2.5-year period (see Figure S3).

To obtain a more reliable estimate of Q_{SS}, we scaled the modeled Q_{SS} through applying a correction factor of 0.56 based on the ratio between the observed and modelled cumulative Q_{SS}. Thus, the scaled model yielded exactly correct cumulative Q_{SS} and acceptably predicted the monthly variation in Q_{SS} (Table S1; Figure S3). The mean relative errors

(55%) were influenced by the notable over-prediction during the very dry months. We acknowledge that the scaled Q_{SS} lacks a separate validation period but considered this acceptable because the purpose of the modelling was to obtain the most reliable estimate of the total transported Q_{SS} over the 9-year period and not to use the model for any scenario simulations.

Table S1 Performance indices of the WSFS-VEMALA for modelling discharge and suspended sediment load in Nov 2009–April 2012 (i.e., from ~4 months before to 2 years after the TSC construction).

Variable	Mean absolute error MAE	Mean relative error MRE	NSE	RMSE	Cumulative error CE	Relative cumulative error RCE
Monthly Q, pre-calibrated	0.038 (m^3/s)	73%	0.91	0.052 (m^3/s)	0.017 (m^3/s)	12.5%
Monthly Q_{TP}, pre-calibrated	7.2 (kg/month)	210%	0.93	9.9 (kg/month)	28 (kg)	3.6%
Daily Q_{TN}, pre-calibrated	4.1 (kg/d)	24%	0.92	12.3 (kg/d)	-29 (kg)	-5.4%
Daily Q_{TOC}, pre-calibrated	19.1 (kg/d)	18%	0.98	31 (kg/d)	151 (kg)	6.7%
Monthly Q_{SS}, pre-calibrated	9.9 (t/month)	77%	-0.18	18 (t)	240 (t)	79%
Monthly Q_{SS}, calibrated	3.01 (t/month)	55%	0.92	4.7 (t/month)	0 (t)	0%

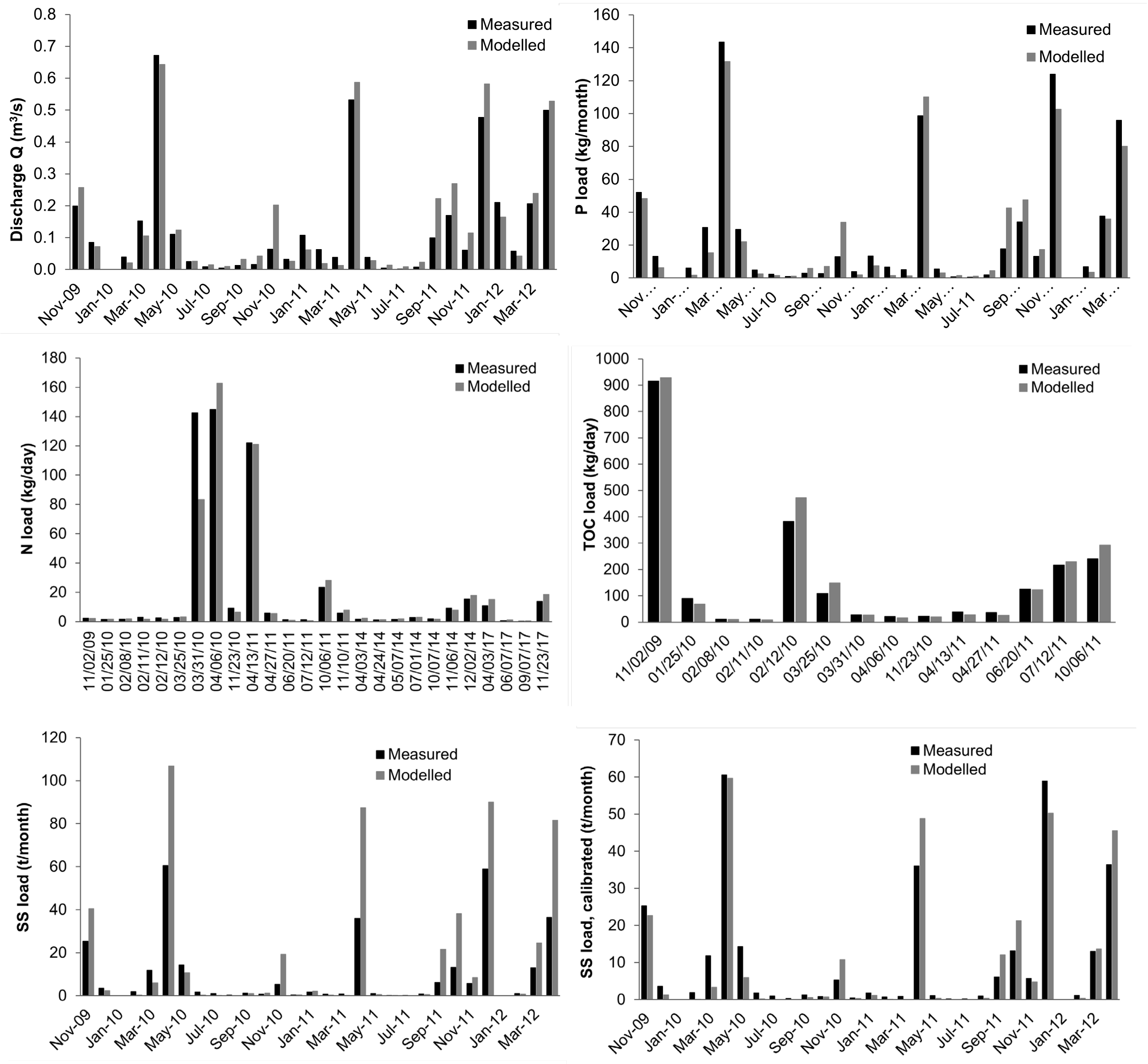

Figure S3 Measured and modelled discharges and loads. Q_{SS} data excluded in Jan 2010 and Jan 2012 and Q data in Jan 2010 because of gaps and disturbances in observational data. For Q_{SS}, the model performance is also shown after calibration of the model through scaling.

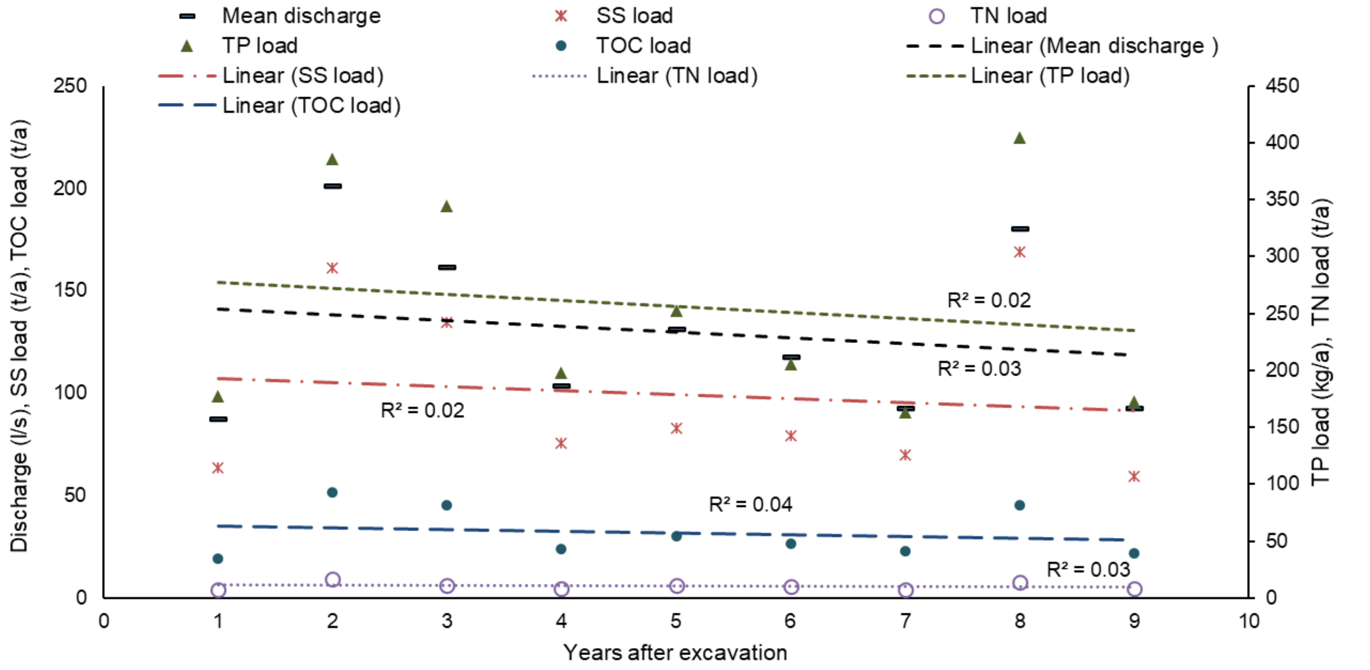

Figure S4 Annual mean discharge, and annual total loads of suspended sediment (Q_{SS}), total phosphorus (Q_{TP}), total nitrogen (Q_{TN}), and total organic carbon (Q_{TOC}) over the 9-year study period.

Text S2 Differences in total and floodplain flow properties between t=0–2 yrs and t=2–9 yrs

The key floodplain flow properties were estimated to enable comparing the two time periods (t=0–2 yrs and t=2–9 yrs) over which the channel morphology development was measured (Table S2). Daily discharges were obtained from the catchment-scale hydrological model WSFS-Vemala (see text S1). Bankful discharge of the low-flow channel was estimated for the geometries representing t=0, t=2 and t=9 yrs based on the common Manning equation (Eq. 2 in [3]), using the mean Manning coefficient n=0.10 observed for the low-flow channel[3]. The hydraulic radius and mean velocity of the Manning equation were defined as the average over the three distinct clusters of the sediment cores. Bankful discharge was interpolated for the years in between using a polynomial regression equation (r^2=1.000).

The inundation duration was determined as the number of days per year on which the daily discharge exceeded the bankful discharge. Inundation frequency was determined as the number of distinct floodplain flow events per year. Daily floodplain discharges for the period t=0–2 yrs were estimated from the daily total discharges using the ratio between these (Table S2) derived for the same channel at t=2 yrs based on the validated two-layer hydraulic model considering the properties of the naturally established grassy floodplain vegetation[6]. The two-layer model (i.e., equations 7 and 8 as reported in[6] and modified from[7]) is based on computing the mean flow velocities in the vegetated parts of the cross-section (u_v) and in the open, unvegetated parts of the cross-section (u_0).

We did not record the vegetation properties at t=2–9 yrs, but the vegetation density increased from t=0–2 yrs based on visual observations. According to the two-layer model, floodplain flow velocity scales with vegetation density (a) as $u_v \sim\sqrt{1/a}$. Thus, using the rather conservative estimate of 30% increase in vegetation density from t=0–2 yrs to t=2–9 yrs leads to 12% lower FP discharge ratio at t=2–9 yrs (Table S2).

The main reasons for the decreased inundation duration and floodplain discharge between t=0–2 yrs to t=2–9 yrs (Table S2) are the progressive enlargement of the cross-sectional area of the low-flow channel and the increased elevation of the floodplain, including the formation of a low levee near the main channel interface (Figure 1; Table 1). These factors increased the bankful discharge by 27%. Secondly, the annual mean discharge decreased by 13% from t=0–2 yrs to t=2–9 yrs, resulting in an overall lower share of total discharge conveyed at flow depths exceeding the floodplain level.

There are minor spatial differences in the floodplain flow properties between the study cross-sections. Based on previous work at the site (e.g., Fig 8 from [6]), up to ~0.1 m lower floodplain water depths were obtained at the upstream end of the study reach compared to the downstream at t=2 years when different sections were initially sown with

different types of plants. Thus, also inundation duration and frequency are expected to have been somewhat lower at the upstream end at *t*=0–2 yrs. The present 9-year monitoring corresponds to the sections with initially sown grassy plants (Grasses-U, Grasses-D) and naturally developed grassy vegetation (Grasses-N). High natural grassy vegetation (Grasses-N) replaced the initially sown plants in the other sections at *t*~2–3 yrs (see details in the beginning of Section 2.2), so that vegetation properties and thus flow resistance became more uniform along the reach, potentially leading to lower differences between the upstream and downstream floodplain water depths and floodplain discharges at *t*=2–9 yrs compared to *t*=0–2 yrs.

Table S2 Total and floodplain flow properties in the two periods for which morphological change was measured.

Flow property	*t*=0–2 yrs	*t*=2–9 yrs	Temporal change (%)
Inundation duration (d/a)	110	97	-11
Inundation frequency (-/a)	12	14	19
Floodplain discharge (10^5 m^3/a)	3.10	2.15	-31
Annual floodplain discharge (m^3/s)	0.010	0.007	-31
Annual mean discharge (m^3/s)	0.144	0.125	-13
Bankful discharge (m^3/s)	0.098	0.124	27
Total discharge above FP level (10^5 m^3/a)	39	31	-21
Ratio of FP discharge to the total discharge (-)	0.08	0.07	-12

Text S3 Accuracy of repeated measurements of cross-sectional geometry

The *t*=0 (2010) and *t*=2 yrs (2012) surveys were conducted with a plummet having a conical tip of a ~2 cm bottom diameter to minimize the penetration into the soft sediments. The *t*=9 yrs (2019) data were collected with a measurement rod having a typical upside down conical tip.

Estimating the vertical accuracy of the elevation surveys in the fully and nearly saturated main channel measurement points is difficult because of the fluffy deposits accumulated after the earlier conventional dredging present particularly at *t*=0–2 yrs[3]. At *t*=0–2 yrs, a maximum of ~3 cm penetration into the saturated main channel bed sediment was estimated based on expert judgement. The upside down conical tip used at *t*=9 yrs had a generally higher tendency for penetration, but the surface sediment layers appeared more consolidated at *t*=9 yrs than at *t*=0–2 yrs because of the re-suspension of the fluffier deposits (see Figure 1 and Table 1), which decreased the tendency for penetration at *t*=9 yrs. Overall, the penetration into the main channel sediment was assumed comparable in different measurement times and was neglected in calculating the net erosion.

For the drier floodplain and banks, the measurement accuracy at *t*=0–2 yrs was ± 6 mm without any systematic error[3]. At *t*=9 yrs, the results on banks and floodplain may be negatively biased by ~5 mm because the upside-down conical tip penetrated up to 5 mm into the non-saturated soil.

Text S4 Processing of the sediment sample data

The electrical mill-grinding was compared to manual grinding with a pestle and mortar to obtain correction factors for converting the concentrations of the manually ground samples to correspond to the electrically ground samples. The correction factors were 1.01 for [P], 1.03 for [S], 1.05 for [N] and 1.19 for [C] based on 5–7 non-sieved floodplain samples; these factors were used for the non-sieved floodplain and bank samples and for the <63 µm fraction of all the sieved test cores because the <63 µm fraction constituted on average over 90% of the total sample mass. The correction factors were 0.96 for [P] and 1.05 for [S] based on 4 non-sieved main channel samples; these factors were used for the non-sieved main channel samples (sample no 27). No >63 µm fractions were manually ground apart from the preliminary analyses.

We determined the repeatability of the elemental analyses based on computing the coefficient of variation (c_v, defined as standard deviation divided by the mean) for the concentrations of replicate samples. 3 samples (<63 µm fractions) with 2 replicates were used for [P] and [S], and 5 samples (4 non-sieved samples and 1 <63 µm fraction) with 2–3 replicates for [C] and [N]. The mean c_v was 1.4% for [P], 1.3% for [S], 2.2% for [N] and 2.6% for [C]. The electrical grinding appeared to produce more homogeneous samples (lower c_v) particularly for the coarser fraction, as shown by the 5 replicate analyses of [C] of the >63 um fractions of 3 samples (numbers 26, 28, 32) exposed to

electrical (mean c_v=16.1%) and manual grinding (mean c_v=28.1%). c_v of repeated samples was notably lower than the changes in the concentrations with sample depth or coring location.

For those sieved samples for which concentrations were not determined for the >63 µm fraction, the concentrations for <63 µm were assumed to be representative of the concentrations of the entire sample, because the related error was low as the <63 µm fraction constituted on average over 90% of the total sample. For samples for which elementary concentrations were not determined, the concentration was approximated as the mean of those of the upper and lower slice of the cores.

Text S5 Equations for computing phosphorus, nitrogen and carbon retention

Mean annual areal sedimentary retention of phosphorus (P), nitrogen (N), and carbon (C), denoted as m_P, m_N, and m_C, respectively, over the period of *T* years, are calculated as follows for the different scenarios:

Scenarios 1 and 2:

$$m_P = \left(\sum_0^{Z_{const}} P_T - \rho[P_{const}](Z_{const} - \Delta Z)\right)/T$$

$$m_N = \left(\sum_0^{Z_{const}} N_T - \rho[N_{const}](Z_{const} - \Delta Z)\right)/T$$

$$m_C = \left(\sum_0^{Z_{const}} C_T - \rho[C_{const}](Z_{const} - \Delta Z)\right)/T$$

where the subscripts *T* and *0* denote the quantities determined at times *t*=T and *t*=0, respectively. $\sum_0^{Z_{const}} P_T$, $\sum_0^{Z_{const}} N_T$, and $\sum_0^{Z_{const}} C_T$ are the cumulative areal masses of P, N, and C, respectively, between *Z*=0 (sediment surface) and Z_{const}, i.e., the depth where [P_{const}], [N_{const}], and [C_{const}] are reached; ρ is the sediment bulk density; [P_{const}], [N_{const}], and [C_{const}] are the constant background concentrations of P, N, and C, respectively, below Z_{const}; ΔZ is the net deposition between *t*=0 and *t*=T years.

Scenarios 3 and 4:

$$m_P = \rho[P_{const}]\Delta Z/T$$

$$m_N = \rho[N_{const}]\Delta Z/T$$

$$m_C = \rho[C_{const}]\Delta Z/T$$

Scenarios 5 and 6:

$$m_P = \left(\sum_0^{Z_{ref,T}} P_T - \sum_0^{Z_{ref,0}} P_0\right)/T$$

$$m_N = \left(\sum_0^{Z_{ref,T}} N_T - \sum_0^{Z_{ref,0}} N_0\right)/T$$

$$m_C = \left(\sum_0^{Z_{ref,T}} C_T - \sum_0^{Z_{ref,0}} C_0\right)/T$$

where $Z_{ref,0} = \max\{Z_{const,0} ; |\Delta Z - Z_{const,T}|\}$ and $Z_{ref,T} = \max\{|\Delta Z + Z_{const,0}|; Z_{const,T}\}$, where Z_{const} is considered as the average over multiple locations.

Text S6 Site-specific factors in aiding in understanding channel development

As typical for low-lying areas around the northern Baltic Sea, the channel flows through post-glacial iron-sulfide bearing clays (Fig. S1) deposited during anaerobic sedimentary conditions of the early Littorina Sea stage that are raised ~20 m above the present sea level by the post-glacial isostatic land uplift[8]. Thus, sedimentary sulphur (S) content was expected to aid in understanding the deposition and excavation history of the channel[9]. In these settings, [S]>0.2% ([S]>2000 ppm) indicates potential acid-sulfate sediments that are not fully oxidized[10] and thus indicates the level above which channel modification works and riverine era deposits are limited to. Fields along our study reach have the highest class 1 acid sulfate soil with the acid layer starting at 0–1 m depth from the field surface[11].

Deviations from the idealized vertical nutrient profiles of scenarios 1–4 (Section 3.2) were observed for the cores RQ and RD. Core RQ has a depleted surface enrichment, and core RD shows relatively high background values for P at Z<27 cm (Figure 5). These deviations can be explained in terms of the location of each core. Core RQ was sampled downstream from a small side-channel with preferential flow and thus enhanced sediment supply observed on the distal floodplain close to RQ. The side-channel likely discharged coarser sediment, further facilitating deposition, as shown by the higher bulk density of the post-excavation deposits for the sediment core RQ compared to the rest of the floodplain cores (0.59 vs 0.45 g/cm^2). The coarser fractions may dilute the nutrient enrichments (Figure 5). Core RD has high S in the lowest part of the soil column (below 27 cm, Figure 5), indicative of frequent submersion and therefore relatively pristine sulfide- and phosphate-mineral rich Littorina sediments protected from oxidation, resembling the main channel cores (Figure S7). Of the cores sampled on the distal floodplain, core RD was located most downstream and likely had a slightly higher inundation frequency than the cores located more upstream (see text S2).

Regarding the main channel, S-rich Littorina layers ([S]>2000 ppm) were observed on average below the depth of 5 cm (ranging from 0 to 13 cm) at t=9 years. They had come closer to the sediment surface compared to t=0 (at 17 cm) because of erosion in the main channel bed. The Littorina sediments were associated with elevated [N] and [C] compared to the [N_{const}] and [C_{const}] of the oxidized layers, as also reported for other Finnish Littorina soils[12].

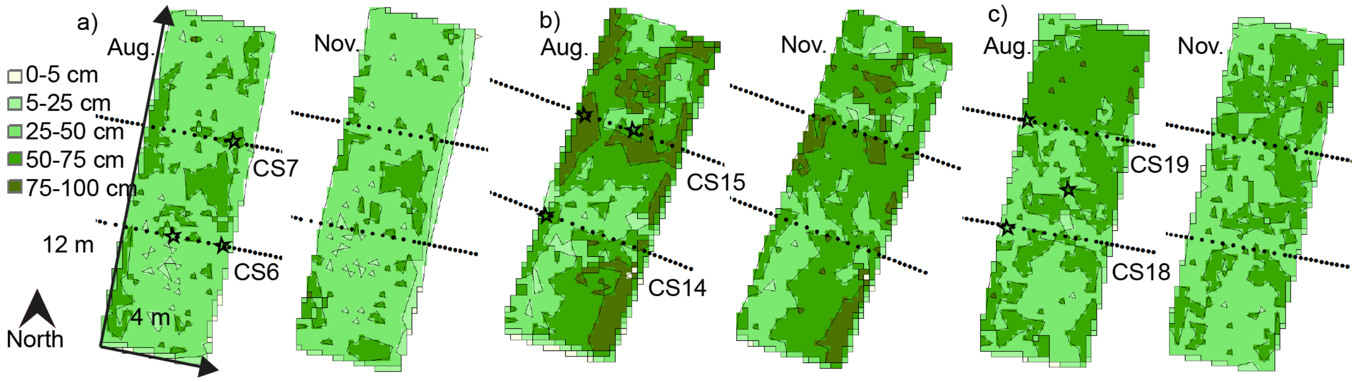

Figure S5 Heights of the grassy floodplain vegetation at and near the sediment core locations (shown with stars) in August conditions at t=2 years (vegetation properties from [13]). Biomass and leaf area index of the vegetation scaled approximately linearly with vegetation height[14]. November vegetation conditions shown for reference.

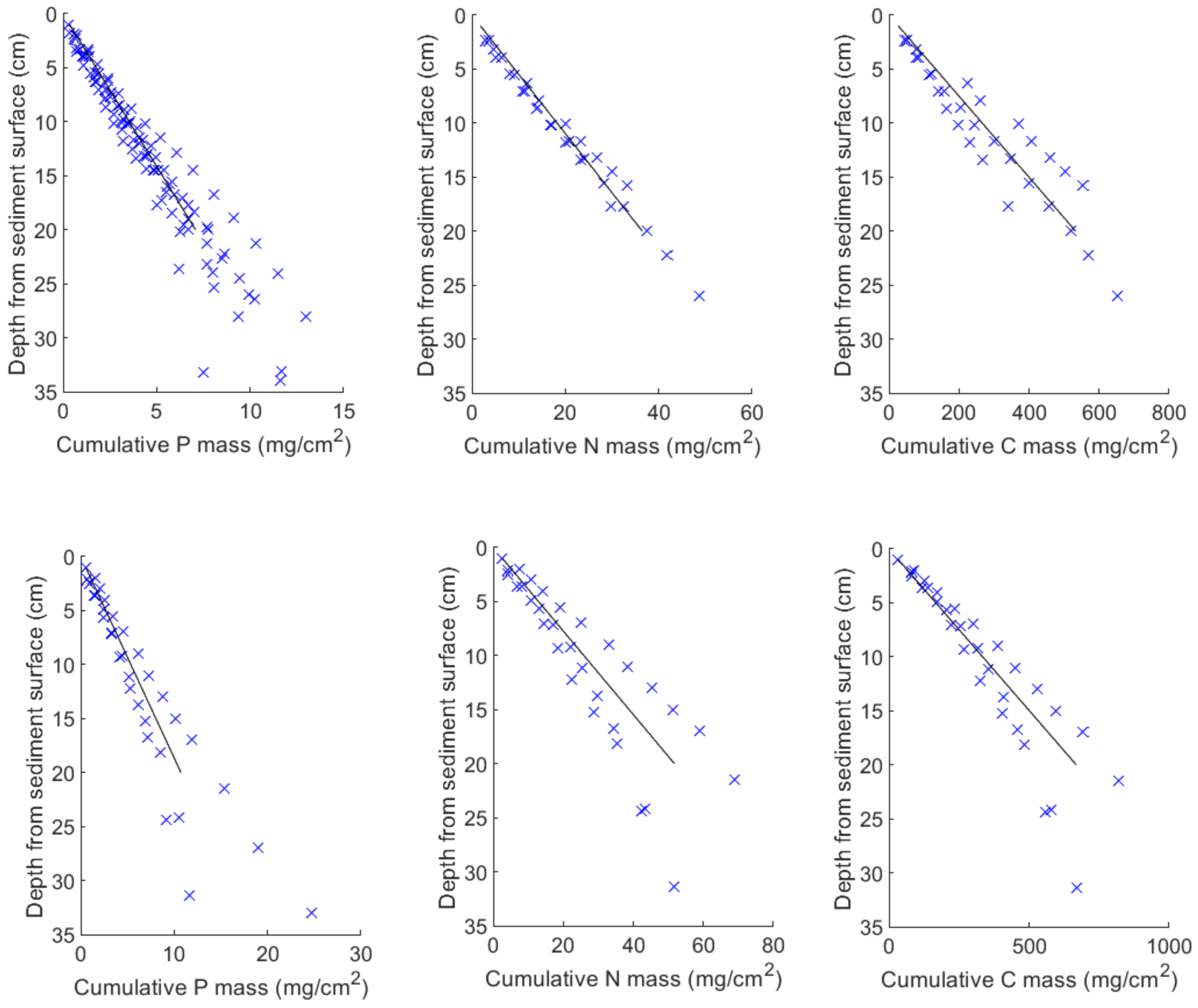

Figure S6 Cumulative masses and the corresponding linear regressions for P, N and C for the floodplain (top row) and banks (bottom row). Data below 20 cm were excluded from the regression analysis to allow more accurate estimates as $|\Delta Z|<20$ cm and $Z_{const}<20$ cm. Note different scaling of the x-axis for floodplain and banks.

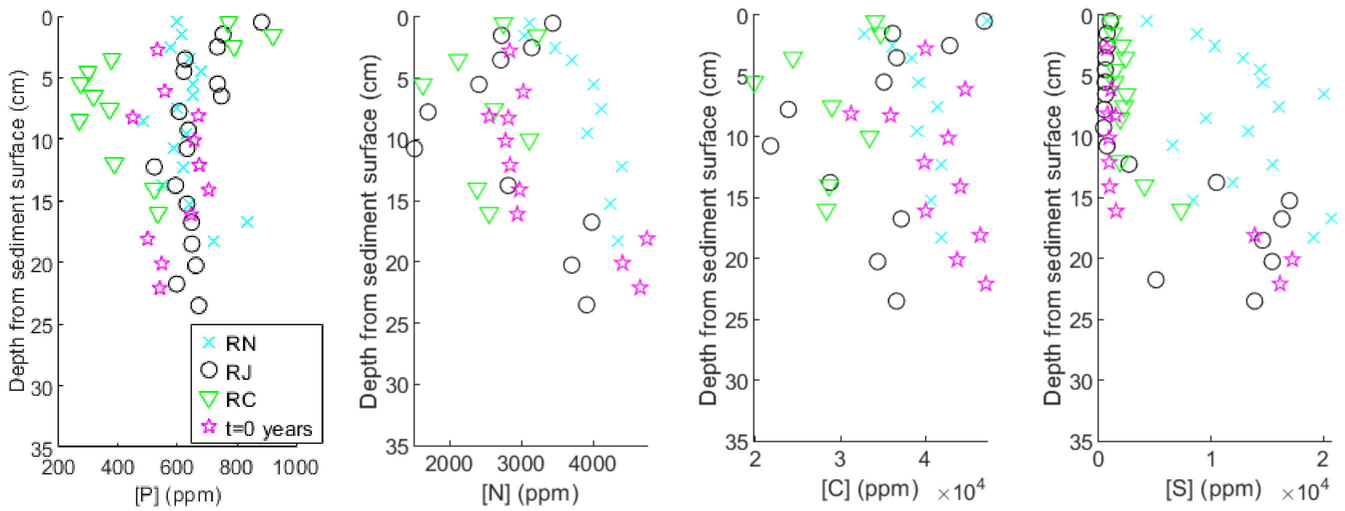

Figure S7 Vertical concentration distributions in the main channel at *t*=9 yrs (cores RN, RJ and RC) and at *t*=0 yrs illustrating the linkages of phosphorus, nitrogen and carbon to sulphur.

References

1. Huttunen, I. *et al.* A National-Scale Nutrient Loading Model for Finnish Watersheds—VEMALA. *Environ. Model. Assess.* **21**, 83–109 (2016).
2. Korppoo, M., Huttunen, M., Huttunen, I., Piirainen, V. & Vehviläinen, B. Simulation of bioavailable phosphorus and nitrogen loading in an agricultural river basin in Finland using VEMALA v.3. *J. Hydrol.* **549**, 363–373 (2017).
3. Västilä, K., Järvelä, J. & Koivusalo, H. Flow–Vegetation–Sediment Interaction in a Cohesive Compound Channel. *J. Hydraul. Eng.* **142**, 04015034 (2016).
4. Västilä, K. *et al.* Agricultural Water Management Using Two-Stage Channels: Performance and Policy Recommendations Based on Northern European Experiences. *Sustainability* **13**, 9349 (2021).
5. Kämäri, M., Tarvainen, M., Kotamäki, N. & Tattari, S. High-frequency measured turbidity as a surrogate for phosphorus in boreal zone rivers: appropriate options and critical situations. *Environ. Monit. Assess.* **192**, 366 (2020).
6. Västilä, K. & Järvelä, J. Characterizing natural riparian vegetation for modeling of flow and suspended sediment transport. *J. Soils Sediments* **18**, 3114–3130 (2018).
7. Luhar, M. & Nepf, H. M. From the blade scale to the reach scale: A characterization of aquatic vegetative drag. *Adv. Water Resour.* **51**, 305–316 (2013).

8. Yli-Halla, M. Acid sulfate soils: A challenge for environmental sustainability. *Ann. Acad. Sci. Fenn.* **1**, 124–141 (2022).

9. Boman, A., Fröjdö, S., Backlund, K. & Åström, M. E. Impact of isostatic land uplift and artificial drainage on oxidation of brackish-water sediments rich in metastable iron sulfide. *Geochim. Cosmochim. Acta* **74**, 1268–1281 (2010).

10. Edén, P. *et al.* Definition and classification of Finnish Acid Sulfate Soils. *Proc. Vol. Geol. Surv. Finl. Guide 56* **56**, 29–30 (2012).

11. Geological Survey of Finland. Spatial data products: Acid sulfate soils 1:250 000, version 1.0. (2013).

12. Paasonen-Kivekäs, M. & Yli-Halla, M. A comparison of nitrogen and carbon reserves in acid sulphate and non acid sulphate soils in western Finland. *Agric. Food Sci.* **14**, 57–69 (2005).

13. Jalonen, J., Järvelä, J., Koivusalo, H. & Hyyppä, H. Deriving Floodplain Topography and Vegetation Characteristics for Hydraulic Engineering Applications by Means of Terrestrial Laser Scanning. *J. Hydraul. Eng.* **140**, 04014056 (2014).

14. Jalonen, J. *et al.* Determining Characteristic Vegetation Areas by Terrestrial Laser Scanning for Floodplain Flow Modeling. *Water* **7**, 420–437 (2015).
